# Comprehensive analysis and validation of novel immune and vascular remodeling related genes signature associated with drug interactions in pulmonary arterial hypertension

**DOI:** 10.3389/fgene.2022.922213

**Published:** 2022-09-06

**Authors:** Jie Wang, Md. Nazim Uddin, Rui Wang, Yue-hong Gong, Yun Wu

**Affiliations:** ^1^ Department of Pharmacy, First Affiliated Hospital of Xinjiang Medical University, Urumqi, China; ^2^ Institute of Food Science and Technology, Bangladesh Council of Scientific and Industrial Research (BCSIR), Dhaka, Bangladesh; ^3^ Department of General Medicine, First Affiliated Hospital of Xinjiang Medical University, Urumqi, China

**Keywords:** pulmonary arterial hypertension, immune-regulation, hypoxia, vascular remodeling, MCT-induced PAH rats’ model

## Abstract

**Background:** Previous studies revealed that the gene signatures are associated with the modulation and pathogenesis of pulmonary arterial hypertension (PAH). However, identifying critical transcriptional signatures in the blood of PAH patients remains lacking.

**Methods:** The differentially expressed transcriptional signatures in the blood of PAH patients were identified by a meta-analysis from four microarray datasets. Then we investigated the enrichment of gene ontology and KEGG pathways and identified top hub genes. Besides, we investigated the correlation of crucial hub genes with immune infiltrations, hallmark gene sets, and blood vessel remodeling genes. Furthermore, we investigated the diagnostic efficacy of essential hub genes and their expression validation in an independent cohort of PAH, and we validate the expression level of hub genes in monocrotaline (MCT) induced PAH rats’ model. Finally, we have identified the FDA-approved drugs that target the hub genes and their molecular docking.

**Results:** We found 1,216 differentially expressed genes (DEGs), including 521 up-regulated and 695 down-regulated genes, in the blood of the PAH patients. The up-regulated DEGs are significantly associated with the enrichment of KEGG pathways mainly involved with immune regulation, cellular signaling, and metabolisms. We identified 13 master transcriptional regulators targeting the dysregulated genes in PAH. The STRING-based investigation identified the function of hub genes associated with multiple immune-related pathways in PAH. The expression levels of RPS27A, MAPK1, STAT1, RPS6, FBL, RPS3, RPS2, and GART are positively correlated with ssGSEA scores of various immune cells as positively correlated with the hallmark of oxidative stress. Besides, we found that these hub genes also regulate the vascular remodeling in PAH. Furthermore, the expression levels of identified hub genes showed good diagnostic efficacy in the blood of PAH, and we validated most of the hub genes are consistently dysregulated in an independent PAH cohort. Validation of hub genes expression level in the monocrotaline (MCT)-induced lung tissue of rats with PAH revealed that 5 screened hub genes (*MAPK1, STAT1, TLR4, TLR2, GART*) are significantly highly expressed in PAH rats, and 4 screened hub genes (*RPS6, FBL, RPS3, and RPS2*) are substantially lowly expressed in rats with PAH. Finally, we analyzed the interaction of hub proteins and FDA-approved drugs and revealed their molecular docking, and the results showed that MAPK1, TLR4, and GART interact with various drugs with appropriate binding affinity.

**Conclusion:** The identified blood-derived key transcriptional signatures significantly correlate with immune infiltrations, hypoxia, glycolysis, and blood vessel remodeling genes. These findings may provide new insight into the diagnosis and treatment of PAH patients.

## Background

Pulmonary arterial hypertension (PAH) is a rare pulmonary disorder characterized by proliferation and occlusion of small changes in pulmonary arterioles that lead to progressive elevation of pulmonary artery pressure, pulmonary vascular resistance, right ventricular failure, and a high mortality rate in patients (Voelkel et al., 2013; Ma and Chung, 2017). PAH’s primary biological and molecular processes included excess vasoconstriction, vascular remodeling, and microthrombotic events (Groth et al., 2014). Aberrantly changes in smooth muscle cell proliferation, smooth muscle cell hypertrophy, and the deposition of matrix proteins within the media of pulmonary arterial vessels are crucial characteristics for developing the PAH (Lyle et al., 2017). The genetic, epigenetic, and environmental factors are linked to the pathogenesis of PAH (Pullamsetti et al., 2016). The precise gene expression patterns regulate PAH’s cellular identity, morphology, and phenotype (Zhang et al., 2020). Deregulated cellular transcriptomes are associated with reduced mitochondrial metabolism, higher glycolysis rate, hyperproliferation, and prevent small vessel loss in PAH (Yuan et al., 2016). Hui Zhang et al. demonstrated that the aberrant expression of genes involves inflammation, apoptosis resistance, proliferation, and metabolism in PAH (Zhang et al., 2020). Applying the bioinformatics methodology, Qing Li et al. identified critical genes associated with signaling pathways and immunity in PAH (Li et al., 2020). Global proteomics is a promising systems-biology approach for deciphering therapeutic actions and associated mechanisms of PAH (Yao et al., 2018). In idiopathic pulmonary arterial hypertension, deregulated transcriptomes across idiopathic pulmonary arterial hypertension lung cells are associated with apoptosis, epigenetic changes, and extracellular matrix organization (Saygin et al., 2020). Integrative analyses of gene expression profiles revealed that the mitotic cell cycle and microtubule cytoskeleton are critically linked with pulmonary artery hypertension (Luo et al., 2020). A whole-blood RNA signature of PAH is associated with disease severity and correlated with poor clinical outcomes in patients (Rhodes et al., 2020). Blood-derived transcriptomes are related to the signaling pathways and gene ontology involved with the pathogenesis and progression of PAH (Ezenwa et al., 2020). These previous studies show that transcriptomic analysis is crucial to identifying the substantial molecular regulators in PAH patients. However, a meta-analysis of multiple gene expression profiling of blood cells obtained from the PAH patients remains lacking.

Herein, a meta-analysis identified the aberrantly expressed transcriptional signatures in the blood tissue of PAH patients. Then we predicted the TFs and KEGG pathways were significantly associated with the up-regulated and down-regulated genes in PAH, respectively. Also, we identified the critical hub genes and clusters extracted from the original PPI network, and investigated the correlation of crucial hub genes with immune infiltrations and hallmark gene sets, including glycolysis, fatty acid metabolism, and oxidative phosphorylation. Moreover, we investigated the correlation of critical prognostic hub genes with blood vessel remodeling genes in PAH. Finally, we investigated the diagnostic efficacy and expression validation of top hub genes in the blood of PAH patients.

## Methods and materials

### Datasets collection and downloading

We followed the methods structure of Dong-feng Li, Aisikeer Tulahong, Md. Nazim Uddin et al., 2021 (Li et al., 2021) and searched the NCBI gene expression omnibus (GEO) database (https://www.ncbi.nlm.nih.gov/geo/) by using the keywords “pulmonary arterial hypertension,” “arterial hypertension,” “pulmonary hypertension,” “idiopathic pulmonary arterial hypertension,” “blood in pulmonary arterial hypertension,” and “roles of blood in pulmonary arterial hypertension,” and identified four gene expression datasets associated with pulmonary arterial hypertension from a different platform. These gene expression datasets have the following criteria: the dataset was derived from the peripheral blood mononuclear cells, the study organisms must be *Homo sapiens*, the dataset had to contain pulmonary arterial hypertension and normal samples, the sample size is greater than 20, and there are at least 10 control samples. The four datasets that met the screening criteria were GSE19617 (Pendergrass et al., 2010), GSE22356 (Risbano et al., 2010), GSE33463 (Cheadle et al., 2012), and GSE131793 (Elinoff et al., 2020). Besides, based on the PRISMA statement, we included the pulmonary arterial hypertension samples in our study and excluded other samples from the same research. The total number of samples included in the study was 190, including 117 pulmonary hypertension samples and 73 normal samples. The description of each dataset is presented in Table 1. Datasets were normalized by quantile normalization or base-2 log transformation for identifying DEGs. Moreover, we validated the expression levels of the top ten hub genes in an independent cohort (GSE38267, *n* = 41) of the blood of PAH patients (Chesné et al., 2014).

**TABLE 1:** The datasets information and patients’ characteristics included in our study.

Serial number	Accession number	Title	Platform	Samples number	Type and Sex of patients
1	GSE13179	Meta-analysis of Blood Genome-Wide Expression Profiling Studies in Pulmonary Arterial Hypertension	GPL6244	*n* = 20, Control = 10, PAH = 10	Female PAH patients
2	GSE19617	Limited systemic sclerosis patients with PAH show biomarkers of inflammation and vascular injury	GPL6480	*n* = 29, Control = 12, PAH = 17	PAH patients, male = 4, female = 13
3	GSE22356	Altered immune phenotype in peripheral blood cells of patients with scleroderma-associated pulmonary hypertension	GPL570	*n* = 28, Control = 10, PAH = 18	Female PAH patients
4	GSE33463	Erythroid-Specific Transcriptional Changes in PBMCs from Pulmonary Hypertension Patients	GPL6947	(*n* = 113), Control = 41, PAH = 72	PAH patients, Male = 14, female = 58

### Identification of differentially expressed genes by meta-analysis

The NetworkAnalyst (Xia et al., 2015a) online analysis tool was used to perform a meta-analysis of four PAH-associated gene expression profile datasets and thus determine the DEG between PAH and normal samples. The selected datasets were normalized by quantile normalization or base-2 log transformation. We applied quantile normalization for the GSE19617 dataset and base-2 log transformation for the GSE22356, GSE33463, and GSE131793, and the ComBat method was used to remove batch effects from multiple datasets (Johnson et al., 2007). Ultimately, 15,067 common genes were identified by integrating the analysis results of the four datasets through the NetworkAnalyst tool. Furthermore, the “limma” package of the R program was used to screen the DEGs between PAH and normal samples, and Cochran’s combination test was used for the meta-analysis (Xia et al., 2015b). The false discovery rate (FDR) (Benjamini and Hochberg, 1995) was adjusted for multiple tests. This study used a total combined effect size (ES) >0.585 and an FDR-corrected *p*-value <0.05 as thresholds to finalize the DEGs.

### Functional enrichment analysis of differentially expressed genes

We entered the up-regulated and down-regulated DEGs into GSEA software (Subramanian et al., 2005) and analyzed the Gene Ontology (GO) of the gene-set enrichment. Besides, we identified significantly enriched KEGG (Kanehisa et al., 2017) and Reactom (Gillespie et al., 2022) pathways associated with DEGs. We used the Database for Annotation, Visualization, and Integrated Discovery (DAVID, https://david.ncifcrf.gov/) v6.8 (Huang et al., 2009) for analyzing the pathway enrichment. In the functional enrichment analysis, the *p*-value<0.05 was considered significant.

### Identified master transcriptional regulators significantly associated with differentially expressed genes

We used Cytoscape (Shannon et al., 2003) plug-in iRegulon (Janky et al., 2014) to identify the MTRs for the up-regulated and down-regulated DEGs. iRegulon identifies the MTRs that are targeting the significant DEGs. The Cytoscape plug-in iRegulon analysis selected a minimum normalized enrichment score (NES) >3.0 for each identified TFs.

### Identification of hub genes and modular analysis by constructing a protein-protein interaction network of differentially expressed genes

Based on the analysis STRING database (the interaction score is 0.40), we construct a PPI network of screened DEGs(Szklarczyk et al., 2019). Then analyzed and visualized the hub genes in the PPI network using the cytohubba plug-in tool in the Cytoscape software (version 3.8.2) (Chin et al., 2014). In this study, genes adjacent to at least 25 other DEGs were defined as hub genes in the PPI network. Furthermore, we performed the molecular complex detection (MCODE) (plug-in in the Cytoscape software) to detect the significant gene modules from the original PPI network(Bader and Hogue, 2003). Finally, we identified several gene modules based on the following criteria: Node Score Cut-off: 0.2, Haircut: true, K-Core: 2, maximum depth from Seed: 100, and MCODE score >5.0.

### Single-sample gene-set enrichment analysis of gene sets and identifying correlation of immune signatures and hallmark gene sets with top hub genes

We used single-sample gene-set enrichment analysis (ssGSEA) to determine the enrichment fraction of immune cells and marker gene sets for each sample and gene-set pair in PAH samples (Hänzelmann et al., 2013). Besides, we collected the marker gene set of immune signatures (including B cells, immature B cells, CD8^+^ T cells, CD4^+^ regulatory T cells, Th1 cells, Th2 cells, Tfh cells, NK cells, macrophages, neutrophils, pDC, activated dendritic cell, immature dendritic cells, monocytes, smooth muscle cells, eosinophil, and mast cells) for calculating the ssGSEA scores of hub genes in various immune signatures (Tirosh et al., 2016; Aran et al., 2017; Davoli et al., 2017; Liu et al., 2018). Moreover, we further downloaded the hallmark gene sets from GSEA (http://www.gsea-msigdb.org/gsea/index.jsp) and identified the ssGSEA scores of hypoxia, apoptosis, glycolysis, fatty acid metabolism, and oxidative phosphorylation (Subramanian et al., 2005). We then investigated Spearman’s correlation between the ssGSEA scores and hub genes identified using PPI. All marker genes and hallmark gene sets are provided in Supplementary Table S1. The correlation value threshold is set as the absolute value is more than 0.20 and *p*-value<0.05.

### Evaluation of the diagnostic value of screened hub genes

To assess the diagnostic value of the final screened hub genes, we calculated and visualized the receiver operating characteristic (ROC) curves using the “pROC” package of the R program, and the area under the ROC curve (AUC) was used to evaluate the efficacy of key genes for differentiating PAH from normal samples (Robin et al., 2011). The larger the AUC value of a single gene, the greater the ability of that gene to discriminate between PAH and normal samples. In the study, an AUC >0.5 for the hub gene with a *p*_value <0.05 was considered a statistically significant diagnostic factor (Yang et al., 2018).

### Expression validation of key hub genes in an independent cohort dataset

To validate the expression levels of the top ten hub genes, we used an independent cohort (GSE38267, *n* = 41) of blood in PAH patients (Chesné et al., 2014). An interactive web tool, GEO2R (http://www.ncbi.nlm.nih.gov/geo/geo2r), was used to identify the differential expressed genes between two groups. GEO2R tool using the GEOquery and limma R packages from the Bioconductor project (http://www.bioconductor.org/). The thresholds of *p*-value < 0.05 and |LogFC| (fold change) > 0.30 were set to identify the significant level of DEGs. We took the gene with the highest fold change value with a substantial *p*-value for multiple probes of a single gene.

### Correlation analysis between the hub genes and blood vessel remodeling genes

We downloaded the blood vessel remodeling genes from the GSEA (http://www.gsea-msigdb.org/gsea/msigdb/cards/GOBP_BLOOD_VESSEL_REMODELING.html) (Subramanian et al., 2005). Pearson’s rank correlation test was utilized to identify the relationship between top hub genes and blood vessel remodeling genes in the integrated four datasets of PAH patients. We employed the R programming software (Version 4.1.0) to calculate the correlation coefficients, and the *p*-value < 0.05 was considered statistically significant.

### Identification of food and drug administration-approved drug-hub gene interaction and their molecular docking with hub genes

We employed DGIdb to identify the FDA-approved drugs that target the hub genes (Cotto et al., 2018). DGIdb collected the interaction of drug-gene data from 30 disparate sources, including DrugBank, ChEMBL, Ensembl, NCBI Entrez, PharmGKB, PubChem, clinical trial databases, and literature in NCBI PubMed. We have selected the FDA-approved drugs in the list of identified drug-gene interactions. The drug-gene interaction was visualized by using Cytoscape software. After screening FDA-approved drugs, these drugs were subjected to molecular docking analysis. We selected 3 hub proteins [MAPK1 (2Y9Q), TLR4 (3FXI), and GART (1MEN)] that interact with drugs. Proteins were prepared by using Discovery studio (https://3ds.com/products-services/biovia/products). At first, all water molecules and ligands were discarded from the complex proteins. Subsequently, a molecular docking study was conducted using the virtual screening tool PyRx (https://pyrx.sourceforge.io/).

### Validation of hub genes expression in lung tissue of rats with pulmonary arterial hypertension

To further clarify the accurate expression of the screened hub genes in lung tissues, we induced a PAH model in rats using MCT (Monocrotaline, Sigma-Aldrich) 60 mg/kg intraperitoneally on the first day, and the control group was given equal amounts of saline for 4 weeks according to our previous study. Besides, the mean right ventricular pressure (mRVP) and mean pulmonary artery pressure (mPAR) was measured by the right heart catheter method to ensure the establishment of the experimental animal model (Wang et al., 2022). Total RNA was extracted by Trizol solution from rat lung tissue samples (5 each from PAH rats and normal rats), and qPCR was used to determine the expression levels of critical genes in the lung tissues of PAH rats and normal rats. First-strand synthesis was performed using a reverse transcription kit (Foregene, Chengdu, China). Expression levels of essential genes were quantified using SYBR Green Master Mix (SYBRGREEN, Beijing, China). The primers used in the quantitative polymerase chain reaction are listed in Supplementary Table S2. A quantitative RT-polymerase chain reaction was performed on VIIa™ 7 System software (Thermo Fisher Science, ABI7500, United States). The results were normalized to the expression of GAPDH, which showed a fold change (2^−ΔΔCT^).

### Statistical analysis

We employed Pearson’s or Spearman’s correlation test to identify the significant relationship between the two variables. We used Spearman’s correlation test to analyze the relationship between the expression levels of hub genes and the ssGSEA scores of immune signatures and hallmark gene sets because these data were not normally distributed (Uddin et al., 2021). We used Pearson’s correlation test to analyze the gene-gene relationship because these data were normally distributed (Uddin et al., 2021). We utilized the R package “ggplot2” to visualize this study’s data (Gómez Rubio, 2017).

## Results

### Meta-analysis identifying differentially expressed genes in pulmonary arterial hypertension

**FIGURE 1:**
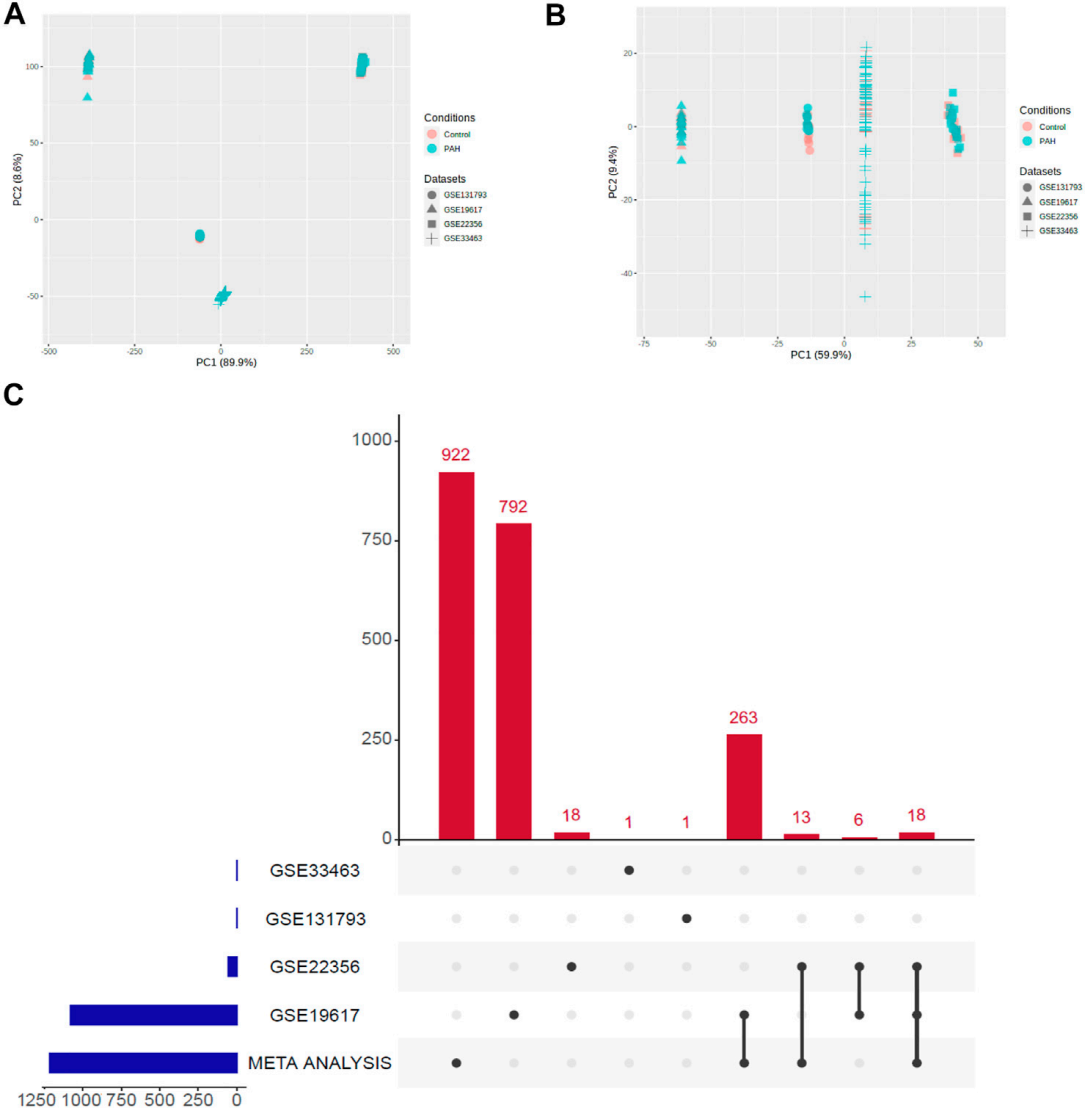
Illustration of PCA and upset plot. **(A)** Before batch effect removal of four different datasets using the ComBat method. **(B)** After batch effect removal of four different datasets using the ComBat method. **(C)** An upset plot of gene sets from meta-analysis versus each of the datasets.

We applied the meta-analysis module of NetworkAnalyst software (https://www.networkanalyst.ca/) to combine the four datasets. We used the ComBat method to remove the batch effect of multiple datasets. The effect of the ComBat method is described in Figures 1A,B. The multidimensional scaling of the datasets demonstrates that before the batch effect adjustment, each dataset is separated from all the others, whereas after batch effect adjustment, samples from all the datasets are dispersed (Figures 1A,B). Meta-analysis more confidently identified the combined Effect size (ES) of DEGs. ES is the difference between two group means divided by standard deviation, considered combinable and comparable across different studies (Xia et al., 2015b). We revealed that the meta-analysis identified more DEGs than the analysis of individual datasets (Figure 1C). Integrated four gene expression datasets identifying 1,216 DEGs, including 521 up-regulated (Supplementary Table S3) and 695 down-regulated (Supplementary Table S4) genes in the peripheral blood cells of patients with PAH. It is worth noting that the top 25 (The highest combined effect size) up-regulated genes, including *NFE2, CYFIP1, PDK4, CTNNA1, MS4A4A, KYNU, CHST15, RBM47, SORT1, GCNT1,* and *ATP6V0A1* (Table 2), which are many of the blood-derived-transcriptomes and have been found associated with the pathogenesis of PAH and cardiovascular diseases (Yuan et al., 2016; Goettsch et al., 2017; Luo et al., 2017). For example, *PDK4*, a gene coding for an enzyme that suppresses mitochondrial activity in favor of glycolysis, was significantly elevated in PAH pericytes and associated with reduced mitochondrial metabolism and higher rates of glycolysis and hyper-proliferation in PAH (Yuan et al., 2016). *CHST15*, another up-regulated gene in blood, is also up-regulated in the pericytes of PAH (Yuan et al., 2016). Evaluated Serum sortilin is associated with lipid metabolism, inflammation, and vascular calcification in the risk of major adverse cerebrovascular and cardiovascular events (Goettsch et al., 2017). The expression levels of ANXA2 contribute to pulmonary microvascular integrity by enabling vascular endothelial cadherin-related phosphatase activity (Luo et al., 2017).

**TABLE 2:** The top 25 up-regulated genes (the highest ES) in the blood cells of PAH relative to normal samples.

Entrez ID	Symbol	Name	Combined ES	*p*-value
4778	*NFE2*	nuclear factor, erythroid 2	1.403	3.99E-04
23191	*CYFIP1*	cytoplasmic FMR1 interacting protein 1	1.390	2.86E-02
5166	*PDK4*	pyruvate dehydrogenase kinase 4	1.351	1.85E-05
1495	*CTNNA1*	catenin alpha 1	1.280	1.63E-02
51338	*MS4A4A*	membrane spanning 4-domains A4A	1.267	1.10E-04
8942	*KYNU*	kynureninase	1.266	6.64E-03
51363	*CHST15*	carbohydrate sulfotransferase 15	1.247	4.69E-04
54502	*RBM47*	RNA binding motif protein 47	1.217	1.23E-02
6272	*SORT1*	sortilin 1	1.210	2.05E-02
2650	*GCNT1*	glucosaminyl (N-acetyl) transferase 1	1.200	1.03E-08
535	*ATP6V0A1*	ATPase H+ transporting V0 subunit a1	1.194	1.85E-02
2358	*FPR2*	formyl peptide receptor 2	1.189	2.15E-04
9123	*SLC16A3*	solute carrier family 16 member 3	1.188	1.03E-04
5321	*PLA2G4A*	phospholipase A2 group IVA	1.181	1.73E-05
54863	*TOR4A*	torsin family 4 member A	1.168	7.62E-04
2720	*GLB1*	galactosidase beta 1	1.167	1.45E-02
56935	*SMC O 4*	single-pass membrane protein with coiled-coil domains 4	1.166	8.96E-06
80896	*NPL*	N-acetylneuraminate pyruvate lyase	1.149	4.54E-03
25801	*GCA*	grancalcin	1.143	2.02E-04
26509	*MYOF*	myoferlin	1.134	7.90E-06
302	*ANXA2*	annexin A2	1.131	7.29E-03
2752	*GLUL*	glutamate-ammonia ligase	1.118	5.54E-09
4066	*LYL1*	LYL1 basic helix-loop-helix family member	1.117	6.40E-08
4005	*LM O 2*	LIM domain only 2	1.117	2.99E-02
3045	*HBD*	hemoglobin subunit delta	1.104	5.54E-09

Also, the top 25 (The lowest combined effect size) down-regulated genes, including *MYLIP, DNAJB1, MGAT4A, PIK3IP1, ARL4C, CAMK4, FCGBP, RORA, HSF2, ZNF256, OXNAD1, DUSP2, PHC3,* and *CAMK2N1* (Table 3)*,* which are the number of the blood-derived down-regulated transcriptomes, linked with the pathogenesis of PAH, lung diseases, and cardiovascular diseases (Lenna et al., 2013; Zhu et al., 2015; Valisno et al., 2021). It was reported that the expression of the *DNAJB1* gene positively correlates with the severity of PAH disease (Lenna et al., 2013). TCF7, a transcription factor, is involved in pulmonary infection, allergy, or asthma through promoting T cells differentiating to Th2 or memory T cells (Zhu et al., 2015). BCL11B is associated with aortic smooth muscle function regulation and is a potential therapeutic target for vascular stiffness and regulating organ damage (Valisno et al., 2021). Altogether, it suggests that the blood cell-derived deregulated transcriptomes are linked with complications of PAH.

**TABLE 3:** The top 25 down-regulated genes (the lowest ES) in the blood cells of patients with PAH relative to normal samples.

Entrez ID	Symbol	Name	Combined ES	*p*-value
29116	*MYLIP*	myosin regulatory light chain interacting protein	−1.484	0.00E+00
3337	*DNAJB1*	DnaJ heat shock protein family (Hsp40) member B1	−1.405	3.42E−08
11320	*MGAT4A*	alpha-1,3-mannosyl-glycoprotein 4-beta-N-acetylglucosaminyltransferase A	−1.404	9.41E-06
113791	*PIK3IP1*	phosphoinositide-3-kinase interacting protein 1	−1.381	1.68E-12
10123	*ARL4C*	ADP ribosylation factor like GTPase 4C	−1.353	2.23E-12
814	*CAMK4*	calcium/calmodulin dependent protein kinase IV	−1.348	3.04E-03
8857	*FCGBP*	Fc fragment of IgG binding protein	−1.344	2.37E-03
6095	*RORA*	RAR related orphan receptor A	−1.329	4.19E-12
3298	*HSF2*	heat shock transcription factor 2	−1.326	2.61E-03
10172	*ZNF256*	zinc finger protein 256	−1.322	3.28E-06
92106	*OXNAD1*	oxidoreductase NAD binding domain containing 1	−1.301	1.58E-05
1844	*DUSP2*	dual specificity phosphatase 2	−1.290	2.01E-11
80012	*PHC3*	polyhomeotic homolog 3	−1.284	1.99E-05
55450	*CAMK2N1*	calcium/calmodulin dependent protein kinase II inhibitor 1	−1.279	2.18E-11
64112	*MOAP1*	modulator of apoptosis 1	−1.273	2.58E-11
6932	*TCF7*	transcription factor 7	−1.241	8.54E-11
64919	*BCL11B*	BAF chromatin remodeling complex subunit BCL11B	−1.226	1.47E-10
81539	*SLC38A1*	solute carrier family 38 member 1	−1.225	1.33E-04
4306	*NR3C2*	nuclear receptor subfamily 3 group C member 2	−1.222	3.70E-07
23348	*DOCK9*	dedicator of cytokinesis 9	−1.204	4.05E-03
7597	*ZBTB25*	zinc finger and BTB domain containing 25	−1.193	2.70E-07
5590	*PRKCZ*	protein kinase C zeta	−1.193	8.84E-05
340542	*BEX5*	brain expressed X-linked 5	−1.184	6.25E-10
79612	*NAA16*	N-alpha-acetyltransferase 16, NatA auxiliary subunit	−1.170	9.47E-10
7695	*ZNF136*	zinc finger protein 136	−1.168	2.70E-07

### Identifying significant gene ontology and pathways associated with blood derived-differentially expressed genes in pulmonary arterial hypertension patients

**FIGURE 2:**
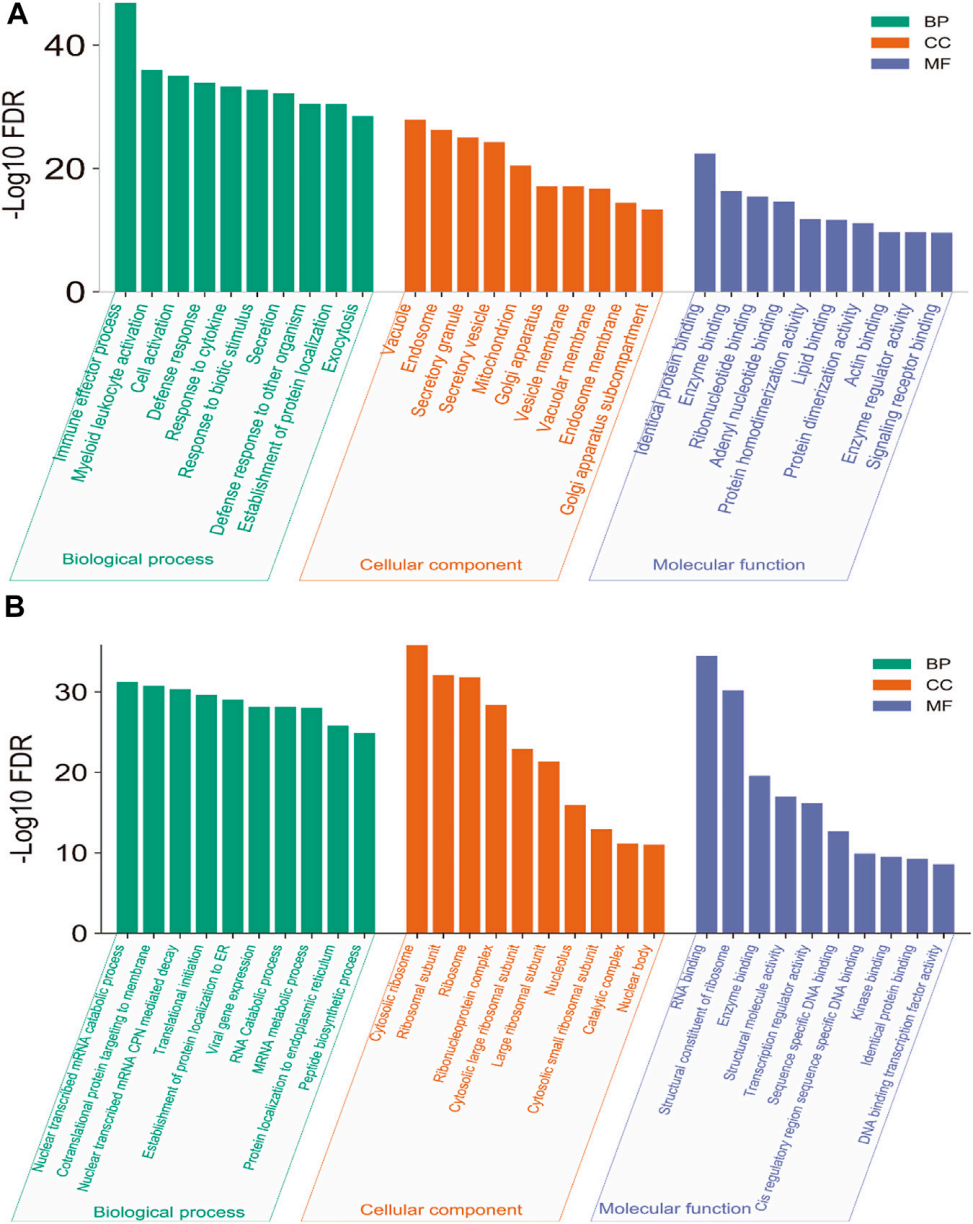
Significantly up-regulated **(A)** and down-regulated **(B)** GO in the blood of PAH.

We investigated the significant (FDR<0.05) Gene Ontology (GO) and KEGG pathways enrichment of blood genes (521 up-regulated genes and 695 down-regulated genes) that are differentially expressed. GO analysis identified the various biological process (BP), cellular components (CC), and molecular function (MF) that are associated with deregulated blood-derived DEGs. We identified different up-regulated BP (Supplementary Table S5 and top ten shown in Figure 2A), including immune effector process, myeloid leukocyte activation, cell activation, defense response, response to cytokine, response to biotic stimulus, secretion, defense response to other organisms, cell activation involved in immune response, myeloid leukocyte mediated immunity, innate immune response, leukocyte mediated immunity, cytokine-mediated signaling pathway, intracellular transport, response to the virus, and defense response to the virus. The up-regulated CCs are mainly involved with membrane-bound cell organelles and cellular membrane (Supplementary Table S6 and Figure 2A), including vacuole, endosome, secretory granule, secretory vesicle, mitochondrion, Golgi apparatus, vesicle membrane, vacuolar membrane, and endosome membrane.

The MF associated with up-regulated DEGs is mainly involved with macromolecular binding capacity, including golgi apparatus sub-compartment, identical protein binding, enzyme binding, ribonucleotide binding, adenyl nucleotide binding, protein homodimerization activity, lipid binding, protein dimerization activity, actin-binding, enzyme regulator activity, and signaling receptor binding (Supplementary Table S7 and Figure 2A). Also, we identified the BP, CC, and MF associated with down-regulated DEGs. The down-regulated GO mainly involves the catabolic process, ribosomal subunit, and molecular binding activity in the cellular system (Supplementary Tables S8–S10, Figure 2B).

**FIGURE 3:**
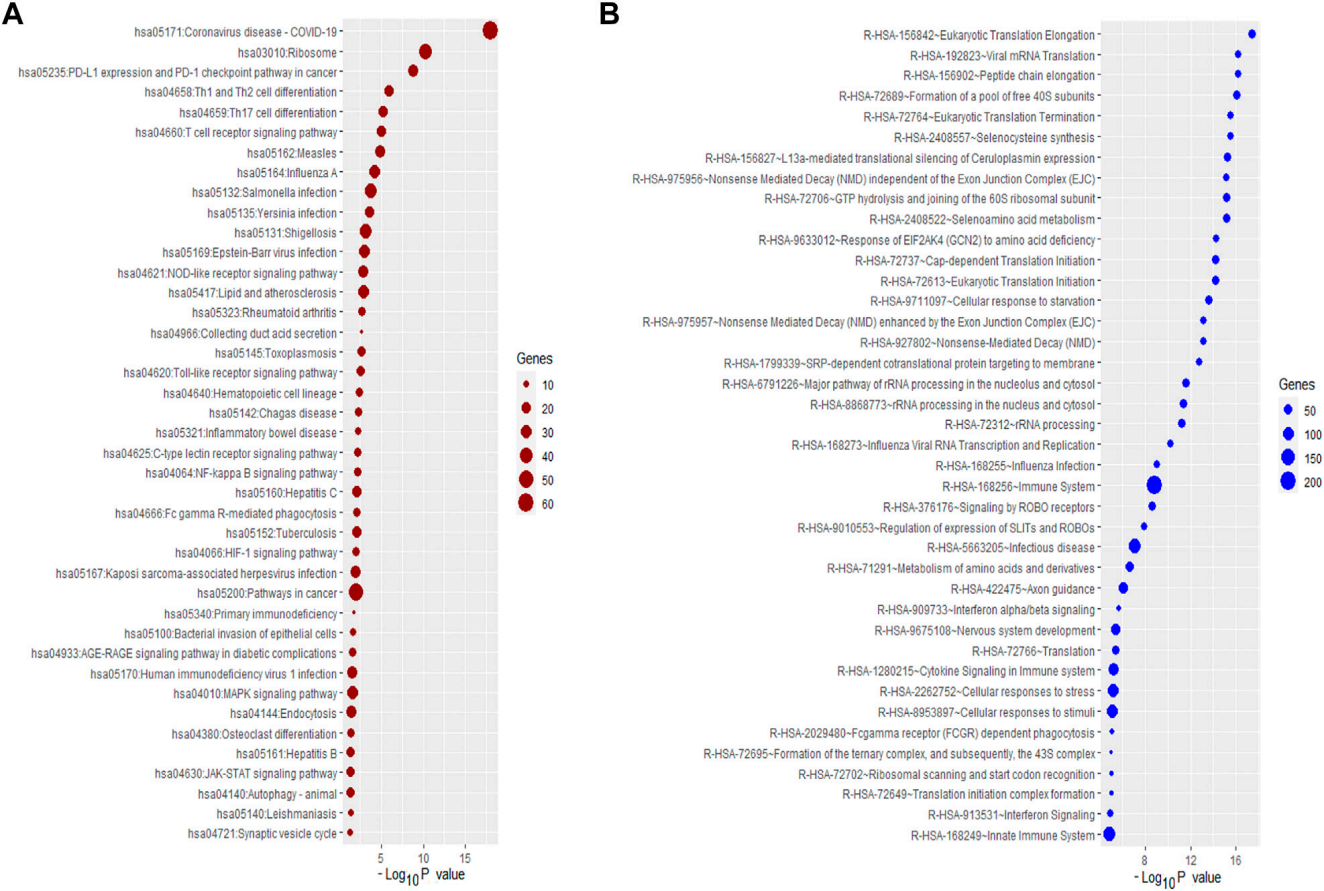
The Significantly enriched pathways associated with DEGs. **(A)** The enriched KEGG pathways. **(B)** The enriched some of the top Reactome pathways.

We analyzed the enrichment of KEGG and Reactome pathways associated with DEGs (Figure 3). The KEGG pathways are significantly associated with immune regulation (such as PD-L1 expression and PD-1 checkpoint pathway in cancer, Th1 and Th2 cell differentiation, Th17 cell differentiation, T cell receptor signaling pathway, T cell receptor signaling pathway, NOD-like receptor signaling pathway, Hematopoietic cell lineage, Fc gamma R-mediated phagocytosis, Primary immunodeficiency, NF-kappa B signaling pathway, Rheumatoid arthritis, and JAK-STAT signaling pathway), cellular signaling (such as Coronavirus disease-COVID-19, Ribosome, Collecting duct acid secretion, C-type lectin receptor signaling pathway, Pathways in cancer, HIF-1 signaling pathway, AGE-RAGE signaling pathway in diabetic complications, MAPK signaling pathway, Osteoclast differentiation, Synaptic vesicle cycle, and Autophagy—animal), and metabolic disorder (such as Lipid and atherosclerosis). The significantly enriched KEGG pathways are provided in Supplementary Table S11 and Figure 3A. Similarly, we revealed that the DEGs are associated with the enrichment of the Reactome pathways, and the results showed that the DEGs are mainly involved with cellular immunity, cellular signaling, and metabolism (Supplementary Table S11 and Figure 3B). Previous studies demonstrated that the deregulation of these pathways is critically associated with pulmonary diseases (Groth et al., 2014; Awad et al., 2016; Lyle et al., 2017; Bhagwani et al., 2020). For example, the toll-like receptor signaling pathway can modulate cell functions of the vascular system for regulating immune function and inflammation (Bhagwani et al., 2020). The Toll-like receptor signaling pathway is enriched within the PAH gene signature (Elinoff et al., 2020). Cytokine-cytokine receptor interaction could stimulate the inflammatory process as a significant contributor to the development of pulmonary hypertension (Groth et al., 2014). MAPK signaling pathway-related proteins, including extracellular signal-regulated kinases 1 and 2 and p38 mitogen-activated protein kinases (MAPKs), play a substantial role in proliferation and cell motility in various PAH experimental models (Awad et al., 2016). Vascular smooth muscle contraction is one of the essential processes in developing PAH (Lyle et al., 2017). The ribosome and base excision repair pathways were the most affected signaling pathways in the PAH after medication (Yao et al., 2018). The vasculature’s RNA signaling correlates with the PAH pathobiology (Turton et al., 2020). Signal transduction pathways such as the T cell receptor signaling pathway and natural killer cell-mediated cytotoxicity are associated with PAH development (Malenfant et al., 2013).

Furthermore, we analyzed the enrichment of KEGG and Reactome pathways associated with up-regulated and down-regulated genes. The up-regulated KEGG pathways are significantly involved with immune regulation signaling, including Toll-like receptor signaling pathway, adipocytokine signaling pathway, Fc gamma R-mediated phagocytosis, *Vibrio cholerae* infection, NOD-like receptor signaling pathway, PD-L1 expression and PD-1 checkpoint pathway in cancer, and RIG-I-like receptor signaling pathway, PPAR signaling pathway. Also, we revealed that significantly enriched Reactome pathways such as immune system, interferon signaling pathway, cytokine signaling in immune system, toll-like receptor signaling pathway, et al., are significantly associated with up-regulated DEGs (Supplementary Table S12). In addition, we analyzed the enrichment of KEGG pathways that are associated with down-regulated genes. Some enriched KEGG pathways are ribosome, T cell receptor signaling pathway, Th1, and Th2 cell differentiation, Th17 cell differentiation, Primary immunodeficiency, Hematopoietic cell lineage, RNA degradation, Base excision repair, and Inositol phosphate metabolism. Also, we revealed the significantly enriched Reactome pathways, including signal transduction, metabolism of proteins, cellular responses to stress, TCR signaling, adaptive immune system, FLT3 Signaling, et al., which are associated with downregulated DEGs (Supplementary Table S13).

### Identification of master transcriptional regulators that are targeting the differentially expressed genes in pulmonary arterial hypertension

**FIGURE 4:**
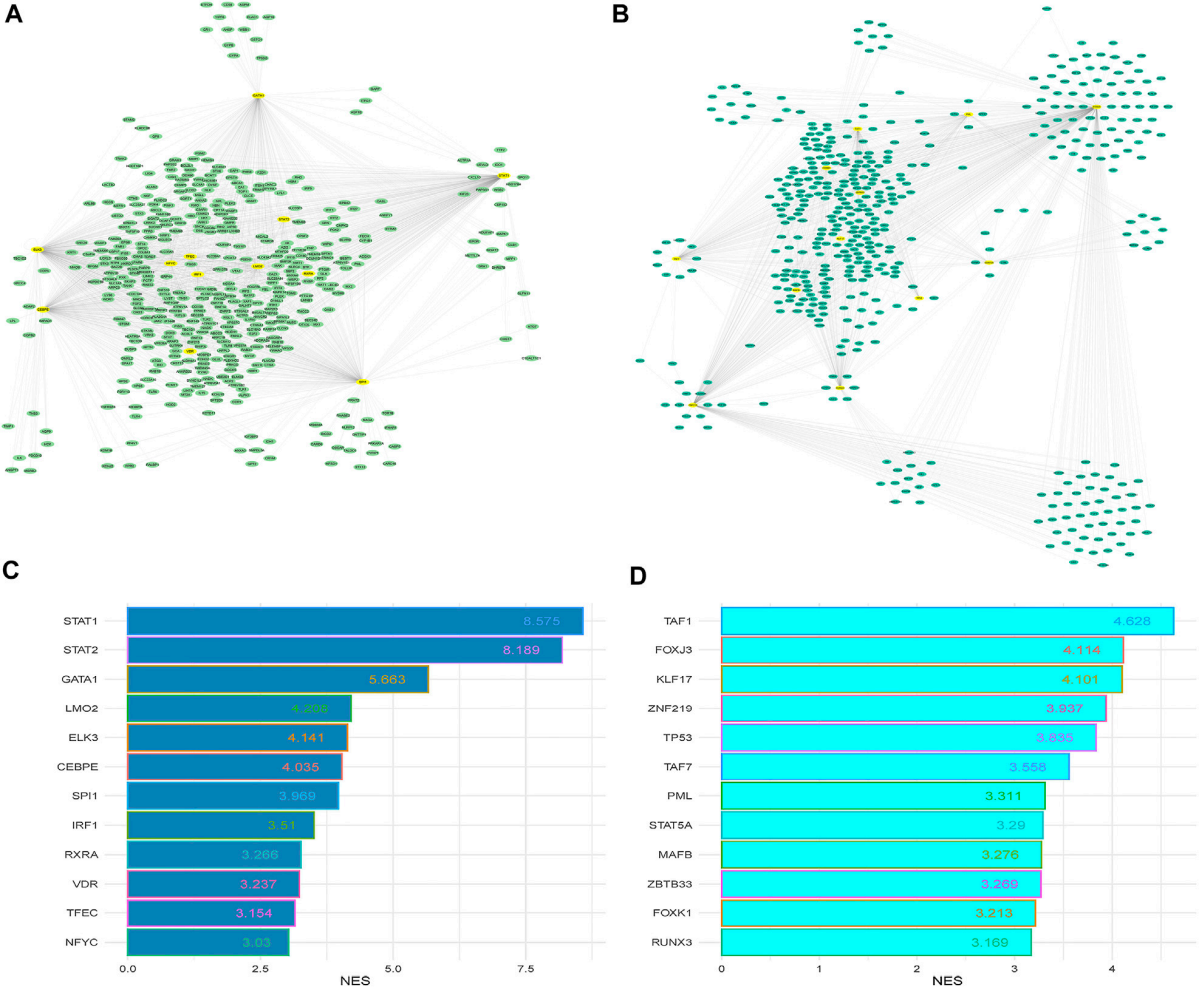
The master transcriptional regulators (MTRs) regulatory networks and their targeted up-regulated and down-regulated DEGs between PAH and control. **(A)** Regulatory network of the MTRs and their targeted up-regulated genes in the PAH group relative to the control. **(B)** Regulatory network of the MTRs and their targeted down-regulated genes in the PAH group relative to the control. **(C)** The MTRs regulate the up-regulated genes with NES>3.0. **(D)** The MTRs regulate the downregulated genes with NES>3.0. The yellow nodes indicate significantly enriched MTRs, and the other color oval indicates identified DEGs.

MTRs are substantial molecular regulators and target the critical genes in the cellular signaling of human diseases (Rosario et al., 2018). We investigated the MTRs targeting the up-regulated and down-regulated genes in PAH, respectively. We identified 12 MTRs (STAT1, STAT2, CEBPE, RXRA, TFEC, ELK3, VDR, NFYC, LMO2, SPI1, GATA1, and IRF1) which are associated with the interaction of up-regulated DEGs in PAH (Figure 4A). Also, we identified 12 MTRs (TAF1, FOXJ3, KLF17, ZNF219, TP53, TAF7, PML, STAT5A, MAFB, ZBTB33, FOXK1, and RUNX3) which are associated with the interaction of down-regulated DEGs in PAH (Figure 4B). In the regulatory networks between MTRs and DEGs, STAT1 interacts with 289 up-regulated genes with the highest NES (8.575), and TAF1 targets the 135 down-regulated genes with the highest NES (4.628). The level of NES of MTRs are illustrated in Figures 4C,D. The list of targeted DEGs with specific MTRs are tabulated in Supplementary Table S14. Interestingly, the 7 MTRs that included CEBPE, ELK3, LMO2, RXRA, STAT1, TFEC, and VDR are also up-regulated DEGs in PAH, and the 4 MTRs that included FOXJ3, FOXK1, RUNX3, and ZNF219 are also down-regulated DEGs in PAH. Recent studies demonstrated that these transcriptional factors encoding genes are aberrantly expressed in PAH. For example, ELK3, a deregulated gene and transcription factor, is also consistently up-regulated in idiopathic PAH (Saygin et al., 2020). In plasma of idiopathic pulmonary arterial hypertension patients, the RARα gene is expressed in cultured human pulmonary artery smooth muscle cells (Preston et al., 2005). Another up-regulated transcription factor, VDR, is identified in vascular cells, which ultimately controls the numerous processes of cardiovascular diseases, including cell proliferation, differentiation, apoptosis, cell adhesion, oxidative stress, angiogenesis, immunomodulation, and anti-inflammation (Callejo Arranz et al., 2020). In methamphetamine-induced chronic lung injury, the expression of RUNX3 is closely related to oxidative stress (Shi et al., 2020). Altogether, transcription factors are critically correlated with the regulation of PAH.

### Construction of protein-protein interaction networks and identification of blood-derived hub genes in pulmonary arterial hypertension

**FIGURE 5:**
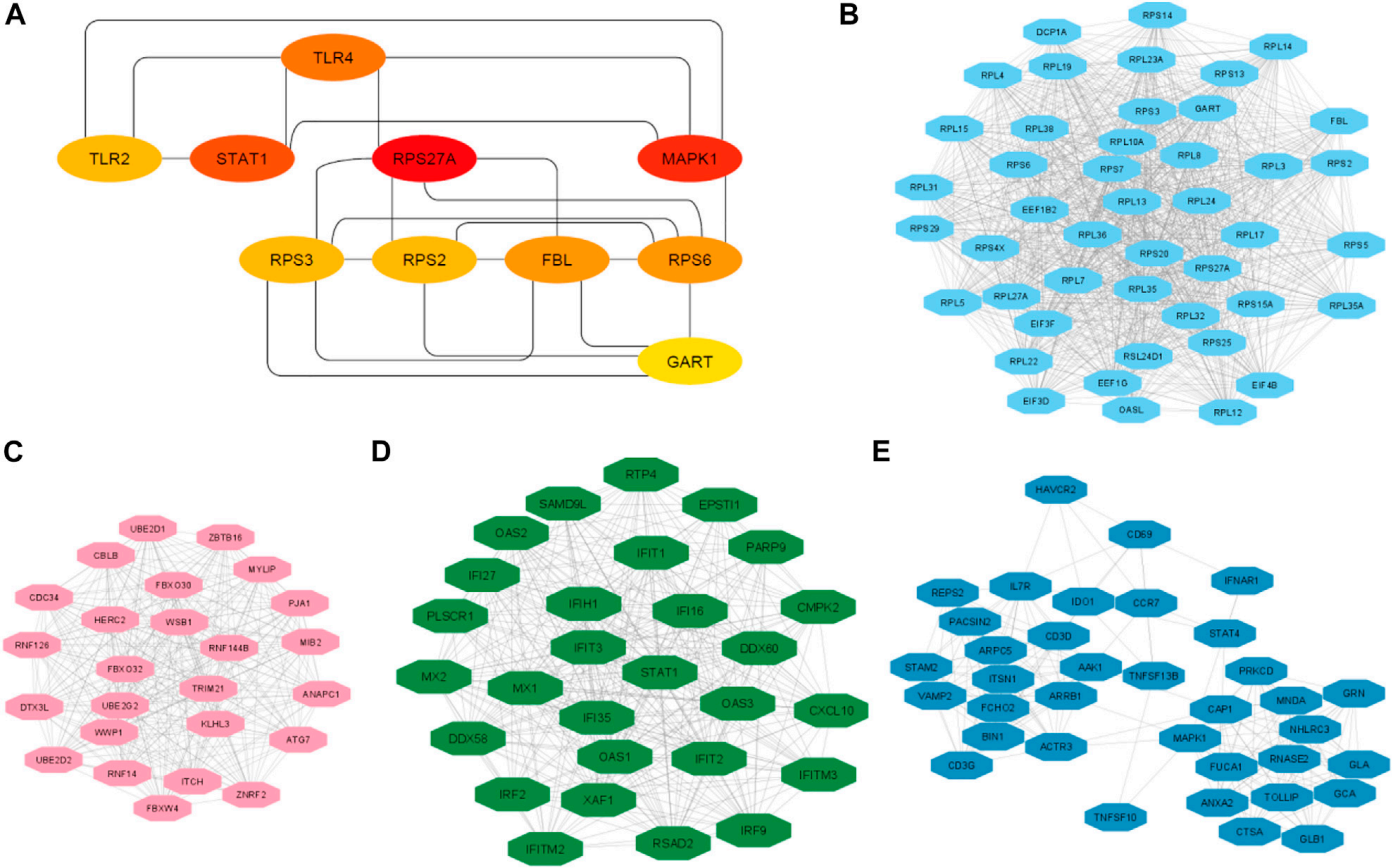
The top ten hub genes and top 4 clusters. **(A)** Identify the top 10 hub genes and their interaction. **(B)** Interaction of nodes in cluster 2. **(C)** Interaction of nodes in cluster 3. **(D)** Interaction of nodes in cluster 3. **(E)** Interaction of nodes in cluster 4.

To investigate the interaction relationship among these identified significant DEGs, the PPI network was constructed using the STRING-based analysis (Szklarczyk et al., 2019). We used Cytoscape plug-in tool cytoHubba to determine the rank of hub genes in the PPI networks (Chin et al., 2014). We inputted all DEGs into the STRING tool and constructed PPI networks. Cytoscape plug-in tool cytoHubba identified the rank of all interacted DEGs based on the degree of interactions. Finally, we got the 197 hub genes (minimum degree of interaction is 25 with other DEGs), including *RPS27A, MAPK1, STAT1, TLR4, RPS6, FBL, TLR2, RPS3, RPS2, GART, OASL, RPS20*, and *CXCL10* (Supplementary Table S15). The interaction of the top ten hub genes is shown in Figure 5. Table 4 describes the differential status of the top ten hub genes with their degree of interaction. The top ten hub genes included five up-regulated genes (*MAPK1, STAT1, TLR4, TLR2,* and *GART*) and five down-regulated genes (*RPS27A, RPS6, FBL, RPS3*, and *RPS2*). The hub gene MAPK1 has interacted with other 103 deregulated genes in PAH. It was reported that mitogen-Activated Protein Kinase (MAPK) inhibition is essential for protecting the lung tissue in PAH (Shafiq et al., 2021). In chimeric mice, TLR4 on platelets contributes to the pathogenesis of pulmonary hypertension (Bauer et al., 2014). Interestingly, ribosomal proteins (*RPS27A, RPS6, RPS3,* and *RPS2*) are down-regulated hub genes, and it was stated by a study that the ribosomal proteins are down-regulated in the PAH of rats (Yao et al., 2018).

**TABLE 4:** The differential status of the top ten hub genes with their degree of interaction.

Rank	Symbol	Name	Differential status	Combined ES	*p* value	Degree of interaction
1	*RPS27A*	Ribosomal protein S27a	Downregulated	−0.643	7.97E-04	148
2	*MAPK1*	Mitogen-activated protein kinase 1	Up-regulated	0.636	9.37E-04	103
3	*STAT1*	Signal transducer and activator of transcription 1	Up-regulated	0.858	5.10E-06	94
4	*TLR4*	Toll-like receptor 4	Up-regulated	0.905	1.34E-06	87
5	*RPS6*	Ribosomal protein S6	Downregulated	−0.762	5.36E-05	75
5	*FBL*	Fibrillarin	Downregulated	−0.970	1.34E-06	75
7	*TLR2*	Toll-like receptor 2	Up-regulated	0.797	2.30E-05	74
7	*RPS3*	Ribosomal protein S3	Downregulated	−0.928	6.42E-07	74
7	*RPS2*	Ribosomal protein S2	Downregulated	−0.649	7.24E-04	74
10	*GART*	Phosphoribosylglycinamide formyltransferase, phosphoribosylglycinamide synthetase, phosphoribosylaminoimidazole synthetase	Up-regulated	0.602	1.92E-03	72

We analyzed the significant MCODE-based cluster analysis of the PPI network derived from all DEGs. The MCODE-based study identified 10 significant clusters from the original PPI networks. The description of MCODE-derived clusters and their associated genes with KEGG pathways are shown in Table 5. The most considerable cluster 1 included 45 DEGs and 956 edges with 43.455 MCODE-based scores (Figure 5A), and the DEGs of cluster 1 are mainly involved with the enrichment of the KEGG ribosome pathway (FDR<0.05). Cluster 2 is associated with ubiquitin-mediated proteolysis. The interactions of nodes in cluster 2 is illustrated in Figure 5B. Interestingly, we found that most of the significant clusters (Cluster 3, cluster 4, cluster 5, cluster 6, cluster 7, cluster 9, and cluster 10) are associated with the enrichment of immune-regulatory pathways, including cytokine-cytokine receptor interaction, Jak-STAT signaling pathway, chemokine signaling pathway, hematopoietic cell lineage, Fc gamma R-mediated phagocytosis, Toll-like receptor signaling pathway, T cell receptor signaling pathway, natural killer cell-mediated cytotoxicity, primary immunodeficiency, Fc epsilon RI signaling pathway, and antigen processing and presentation. The interactions of nodes in cluster 3 and 4 is presented in Figures 5C,D, respectively. It suggests that the PPI-derived significant gene clusters are associated with regulating immunity and other signaling pathways in PAH patients.

**TABLE 5:** MCODE identified 10 significant clusters from the PPI networks of DEGs, and GSEA identified enrichment of KEGG pathways (*p* < 0.05) for a specific gene set of the individual cluster.

Cluster	MCODE Score	Nodes numbers	Edges numbers	Enrichment of KEGG pathways (FDR<0.05)
1	43.455	45	956	Ribosome
2	25	25	300	Ubiquitin mediated proteolysis
3	24.741	28	334	RIG-I-like receptor signaling pathway
4	11.943	36	209	Cytokine-cytokine receptor interaction, Lysosome, Jak-STAT signaling pathway, Chemokine signaling pathway, Hematopoietic cell lineage, Fc gamma R-mediated phagocytosis, Toll-like receptor signaling pathway, T cell receptor signaling pathway, Natural killer cell-mediated cytotoxicity, Primary immunodeficiency, Fc epsilon RI signaling pathway
5	8.129	32	126	Toll-like receptor signaling pathway
6	7.172	30	104	T cell receptor signaling pathway, Hematopoietic cell lineage
				Cytokine-cytokine receptor interaction, Jak-STAT signaling pathway, Primary immunodeficiency, NOD-like receptor signaling pathway, Toll-like receptor signaling pathway, Antigen processing and presentation
7	6.833	13	41	Oxidative phosphorylation
8	6.286	8	22	Base excision repair, Non-homologous end-joining
9	5.5	9	22	Primary immunodeficiency, T cell receptor signaling pathway, Natural killer cell-mediated cytotoxicity
10	5.316	39	101	Endocytosis, Fc gamma R-mediated phagocytosis, Regulation of actin cytoskeleton, Peroxisome

### Hub genes are correlated with the infiltrations of immune cells

**FIGURE 6:**
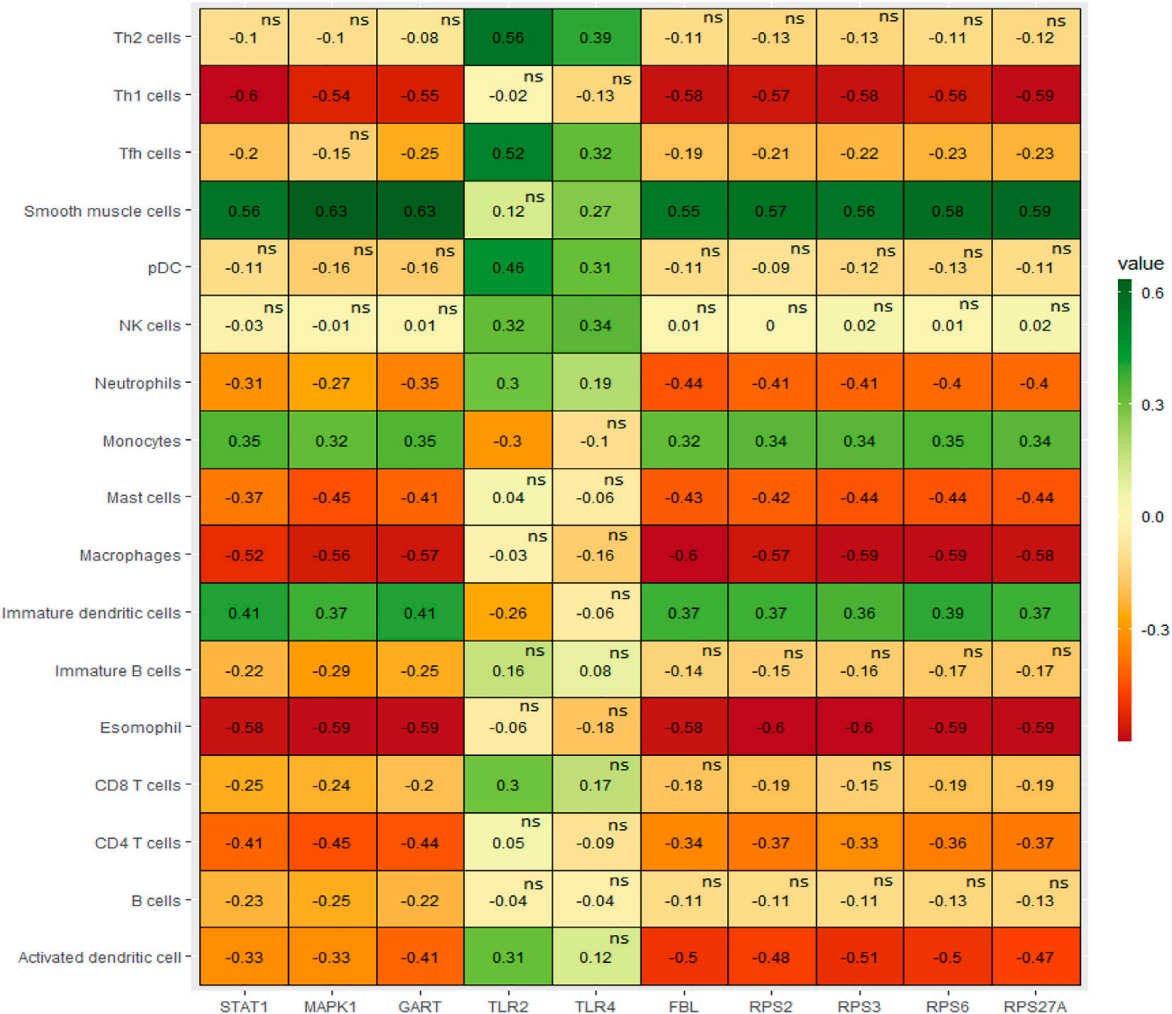
The expression levels of hub genes are significantly correlated with ssGSEA scores of immune cells in PAH (Spearman’s correlation test, *p* < 0.05).

Since the pathogenesis of PAH patients is significantly correlated with immunological responses and inflammations of various inflammatory cells in humans (El Chami and Hassoun, 2012; Rabinovitch et al., 2014; Kim and Choi, 2019), we revealed the relationships between the expressions of the top ten hub genes (*RPS27A, MAPK1, STAT1, TLR4, RPS6, FBL, TLR2, RPS3, RPS2,* and *GART*) and immune infiltrations of various immune cells, the essential immune stimulatory and inhibitory signatures including B cells, immature B cells, CD8^+^ T cells, CD4^+^ regulatory T cells, Th1 cells, Th2 cells, Tfh cells, NK cells, macrophages, neutrophils, pDC, activated dendritic cell, immature dendritic cells, monocytes, smooth muscle cells, esomophil, and mast cells. We found that the expression levels of *RPS27A, MAPK1, STAT1, RPS6, FBL, RPS3, RPS2, and GART* hub genes are positively correlated with ssGSEA scores of immature dendritic cells, monocytes, and smooth muscle cells (Spearman’s correlation test, *p* < 0.05) (Figure 6). In addition, *RPS27A, MAPK1, STAT1, RPS6, FBL, RPS3, RPS2,* and *GART* hub genes are negatively correlated with various immune cells, including pDC, activated dendritic cell, macrophages, neutrophils, CD4^+^ regulatory T cells, Th1 cells, eosinophil, and mast cells (Spearman’s correlation test, *p* < 0.05)**.** The infiltration levels of CD8^+^ T cells are negatively correlated with the expression levels of *RPS27A, MAPK1, STAT1, RPS6, FBL, RPS3,* and *GART* (Spearman’s correlation test, *p* < 0.05)**.** On the other hand, the expression level of the TLR2 hub gene is negatively correlated with monocytes and positively correlated with the infiltrations of various immune cells, including activated dendritic cells, pDC, Tfh cells, and Th2 cells. In addition to the TLR2, the TLR4 hub gene is positively correlated with the infiltrations of pDC and Tfh cells (Spearman’s correlation test, *p* < 0.05)**.** In addition to the correlation of hub genes with immune cells, we revealed that the immune cells are differentiated between the control and PAH patients. Activated dendritic cells, B cells, CD8 T cells, Immature B cells, Macrophages, Mast cells, and Neutrophils are significantly differentiated (t-test, *p* < 0.05) in the integrated four datasets and other individual datasets (Supplementary Table S16). Inflammatory cell infiltration is a prominent characteristic of aberrant vascular remodeling in pulmonary arterial hypertension (PAH) (Elinoff et al., 2020). Suggesting that these immune effector cells are associated with the disease progression. These results indicate that the expression level of hub genes is substantially related to regulating immunity and disease progression in PAH patients.

### Hub genes are correlated with hypoxia and apoptosis

**FIGURE 7:**
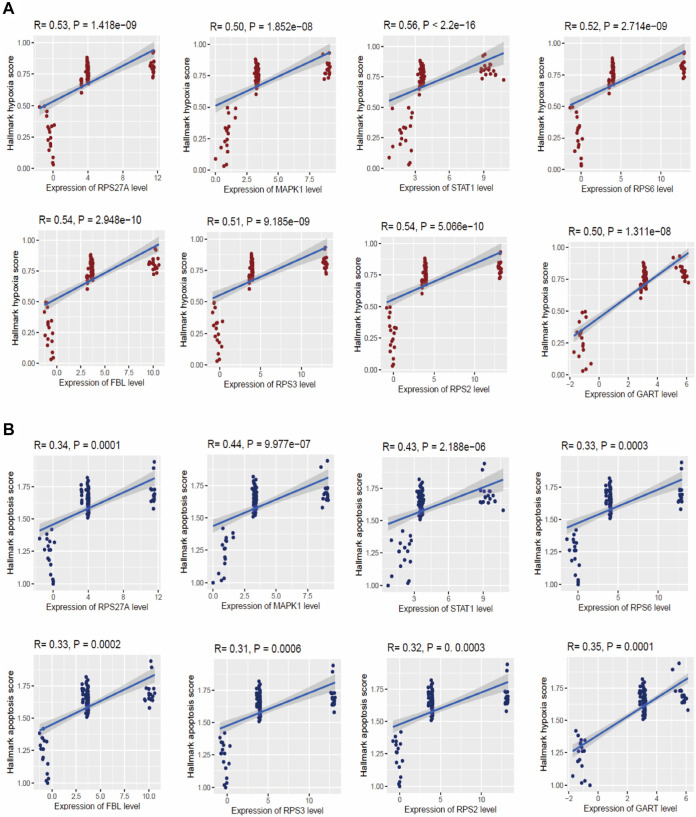
The expression levels of hub genes are significantly correlated with ssGSEA scores of hallmark hypoxia **(A)** and apoptosis **(B)** gene set in PAH (Spearman’s correlation test, *p* < 0.05). The hallmark gene set of hypoxia and apoptosis was downloaded from the GSEA (http://www.gsea-msigdb.org/gsea/index.jsp).

Hypoxia is considered a common cause of several chronic lung diseases, including pulmonary hypertension and pulmonary vascular remodeling (Pak et al., 2007; Voelkel et al., 2013). Therefore, it is crucial to identify the key genes that regulate hypoxia in peripheral blood cells. We investigated the correlation of the hypoxia score (Hallmark hypoxia gene set was downloaded from the GSEA) with the expression levels of the top ten hub genes in four integrated datasets [GSE19617 (Pendergrass et al., 2010), GSE22356 (Risbano et al., 2010), GSE33463 (Cheadle et al., 2012), and GSE131793 (Elinoff et al., 2020)]. Interestingly, we found that the expression of RPS27A, MAPK1, STAT1, RPS6, FBL, RPS3, RPS2, and GART hub genes are positively correlated with hypoxia (ssGSEA score of hallmark hypoxia gene set) in the PAH (Spearman’s correlation test, *p* < 0.05) (Figure 7A). Apoptosis is associated with the initiation of experimental PAH, leading directly to the degeneration of pre-capillary arterioles or selecting hyperproliferative cells that may contribute to “angioproliferative” plexiform lesions (Jurasz et al., 2010). Therefore, we investigated the correlations of significant hub genes with the apoptosis score. Similar to hypoxia, we found that the expression of *RPS27A, MAPK1, STAT1, RPS6, FBL, RPS3, RPS2,* and *GART* hub genes are positively correlated with apoptosis (ssGSEA score of hallmark hypoxia gene set) in the PAH (Spearman’s correlation test, *p* < 0.05) (Figure 7B). The other two hub genes, TLR2 and TLR4, are not significantly correlated with hypoxia and apoptosis in the PAH.

### Hub genes are associated with the metabolic process and oxidative phosphorylation

**FIGURE 8:**
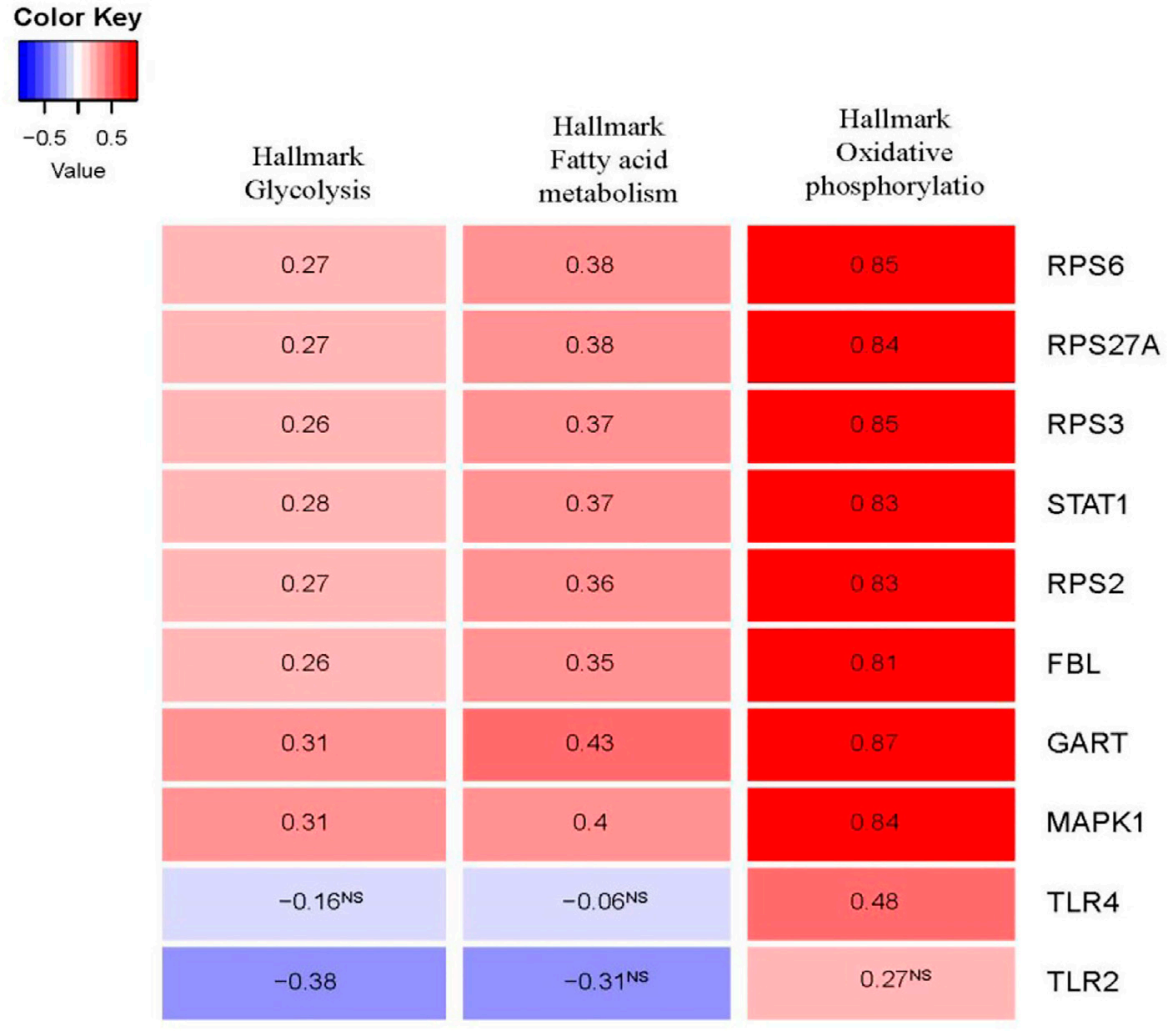
The expression levels of hub genes are significantly correlated with ssGSEA scores of hallmark glycolysis, hallmark fatty acid metabolism, and hallmark oxidative phosphorylation gene set in PAH (Spearman’s correlation test, *p* < 0.05). The hallmark gene sets were downloaded from the GSEA (http://www.gsea-msigdb.org/gsea/index.jsp).

Metabolic aberrations are crucial factors for the disease process of PAH, and these metabolic and mitochondrial dysfunctions are associated with the driving of PAH pathogenesis (Chan and Rubin, 2017; Culley and Chan, 2018). We investigated the correlation of glycolysis, fatty acid metabolism, and oxidative phosphorylation activity with the expression levels of the top ten hub genes in the integrated four datasets (Figure 8). Interestingly, the expression levels of *RPS6, RPS27A, RPS3, STAT1, RPS2, FBL, GART,* and hub genes *MAPK1* are positively correlated with glycolysis, fatty acid metabolism, and oxidative phosphorylation activity (ssGSEA score of hallmark gene sets downloaded from the GSEA) (Spearman’s correlation test, *p* < 0.05). Another hub gene, *TLR2*, is negatively correlated with glycolysis (Spearman’s correlation test, *p* < 0.05).

### Hub genes are correlated with blood vessel remodeling genes

It is widely demonstrated that structural alterations in the vascular wall contribute to all forms of pulmonary hypertension(Shimoda and Laurie, 2013; Tuder, 2017). We speculate that the hub genes are correlated with the expression of blood vessel remodeling genes. To prove our hypothesis, we downloaded the blood vessel remodeling genes from GSEA (http://www.gsea-msigdb.org/gsea/msigdb/cards/GOBP_BLOOD_VESSEL_REMODELING.html) and investigated the correlation of top ten hub genes (*RPS27A, MAPK1, STAT1, TLR4, RPS6, FBL, TLR2, RPS3, RPS2,* and *GART*) with these gene set (Table 6). Interestingly, we found that most of the blood vessels remodeling genes are positively correlated with the expression levels of the top ten hub genes (*RPS27A, MAPK1, STAT1, TLR4, RPS6, FBL, TLR2, RPS3, RPS2,* and *GART*) (Pearson’s correlation test, FDR<0.05)**.** Chesné et al. (2014) discovered the gene signatures highly expressed in the blood of patients with PAH and are associated with inflammation, vascular remodeling, and cell proliferation. It indicates that the expression level of top hub genes is correlated with the blood vessel remodeling genes in PAH.

**TABLE 6:** The blood vessel remodeling genes are positively correlated with the expression levels of the top ten hub genes: *RPS27A, MAPK1, STAT1, TLR4, RPS6, FBL, TLR2, RPS3, RPS2,* and *GART* (Pearson’s correlation test, FDR<0.05).

ID	Symbol	*RPS27A*	*MAPK1*	*STAT1*	*TLR4*	*RPS6*	*FBL*	*TLR2*	*RPS3*	*RPS2*	*GART*
93	*ACVR2B*	0.886	0.831	0.758	0.539	0.859	0.911	0.339	0.853	0.843	0.992
94	*ACVRL1*	0.917	0.866	0.798	0.598	0.894	0.938	0.404	0.887	0.878	0.992
183	*AGT*	0.713	0.639	0.535	0.278	0.674	0.755	0.051^NS^	0.664	0.650	0.933
9474	*ATG5*	0.992	0.974	0.937	0.797	0.985	0.997	0.650	0.983	0.979	0.949
558	*AXL*	0.848	0.792	0.709	0.485	0.819	0.877	0.267	0.811	0.800	0.985
578	*BAK1*	0.988	0.973	0.946	0.810	0.982	0.990	0.662	0.980	0.977	0.935
581	*BAX*	0.987	0.986	0.969	0.861	0.990	0.983	0.772	0.990	0.990	0.874
659	*BMPR2*	0.972	0.942	0.892	0.725	0.958	0.983	0.558	0.954	0.948	0.977
634	*CEACAM1*	0.876	0.820	0.743	0.533	0.848	0.900	0.331	0.840	0.830	0.981
55636	*CHD7*	0.942	0.898	0.839	0.647	0.922	0.959	0.465	0.917	0.909	0.988
23418	*CRB1*	0.819	0.749	0.671	0.450	0.784	0.844	0.241	0.774	0.764	0.952
1471	*CST3*	0.973	0.987	0.989	0.926	0.983	0.959	0.855	0.985	0.989	0.803
54567	*DLL4*	0.986	0.962	0.919	0.775	0.976	0.991	0.624	0.972	0.968	0.953
2034	*EPAS1*	0.755	0.683	0.590	0.350	0.718	0.790	0.111^NS^	0.707	0.694	0.949
2131	*EXT1*	0.943	0.908	0.849	0.662	0.924	0.960	0.481	0.920	0.912	0.987
2255	*FGF10*	0.713	0.631	0.543	0.313	0.671	0.743	0.095^NS^	0.659	0.648	0.893
2316	*FLNA*	0.935	0.964	0.976	0.949	0.952	0.913	0.909	0.957	0.962	0.720
2324	*FLT4*	0.924	0.876	0.810	0.617	0.901	0.943	0.427	0.895	0.887	0.986
2296	*FOXC1*	0.813	0.752	0.660	0.417	0.782	0.847	0.211	0.774	0.762	0.973
2303	*FOXC2*	0.822	0.778	0.691	0.459	0.799	0.852	0.315	0.791	0.785	0.935
3200	*HOXA3*	0.894	0.847	0.771	0.559	0.871	0.917	0.384	0.863	0.856	0.980
3273	*HRG*	0.851	0.787	0.715	0.509	0.819	0.871	0.309	0.809	0.801	0.953
182	*JAG1*	0.825	0.768	0.675	0.444	0.795	0.858	0.240	0.788	0.776	0.976
3976	*LIF*	0.884	0.834	0.763	0.553	0.857	0.906	0.360	0.850	0.842	0.976
4208	*MEF2C*	0.955	0.978	0.987	0.950	0.970	0.936	0.360	0.972	0.977	0.759
8996	*NOL3*	0.816	0.755	0.664	0.435	0.785	0.849	0.211	0.776	0.765	0.978
4846	*NOS3*	0.892	0.837	0.764	0.558	0.866	0.916	0.355	0.859	0.849	0.989
3516	*RBPJ*	0.997	0.993	0.970	0.867	0.997	0.993	0.742	0.996	0.995	0.902
84870	*RSP O 3*	0.613	0.520	0.424	0.188	0.566	0.648	−0.044^NS^	0.552	0.540	0.840
10512	*SEMA3C*	0.819	0.758	0.668	0.442	0.787	0.851	0.221	0.778	0.767	0.975
7040	*TGFB1*	0.925	0.958	0.974	0.952	0.944	0.902	0.921	0.950	0.955	0.699
7052	*TGM2*	0.918	0.876	0.807	0.611	0.895	0.937	0.419	0.890	0.882	0.986
64114	*TMBIM1*	0.975	0.988	0.986	0.928	0.985	0.960	0.851	0.987	0.990	0.804
1636	*ACE*	0.925	0.876	0.812	0.617	0.902	0.944	0.428	0.895	0.888	0.989

Note: NS is non-significant.

### Diagnostic efficacy evaluation of top ten hub genes in integrated datasets of pulmonary arterial hypertension

**FIGURE 9:**
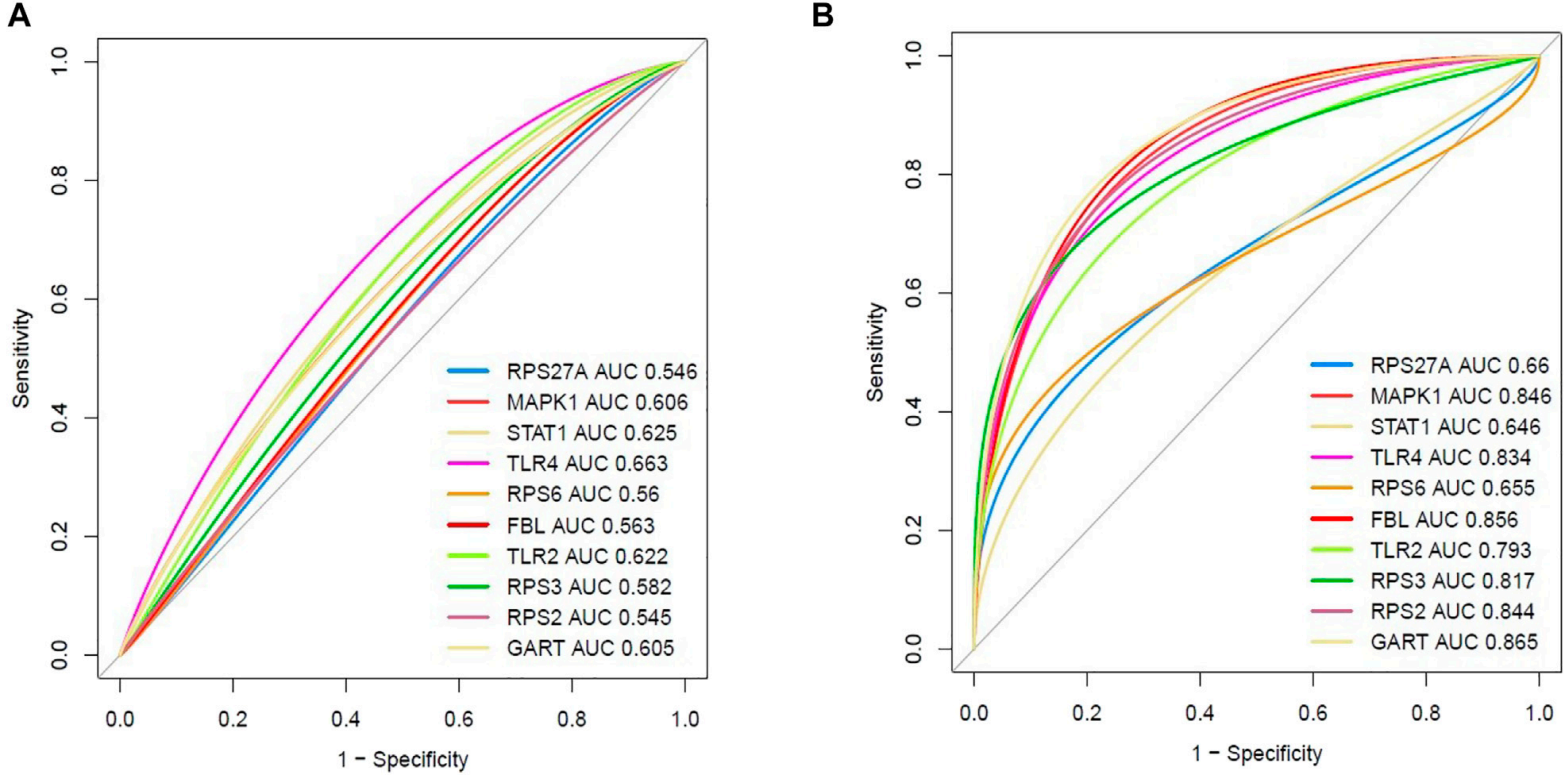
Evaluation of diagnostic efficacy of essential hub genes. **(A)** The receiver operating characteristic (ROC) curve of the top ten hub genes in peripheral blood of PAH patients relative to the normal blood cells (integrated four datasets). **(B)** Validation of the receiver operating characteristic (ROC) curve of the top ten hub genes in peripheral blood (GSE38267).

We speculate that these ten hub genes (*RPS27A, MAPK1, STAT1, TLR4, RPS6, FBL, TLR2, RPS3, RPS2,* and *GART*) have diagnostic value in PAH. We used the integrated data (integrated four datasets) to validate our hypothesis, and the investigated results revealed that the ROC curve of the expression levels of these top ten hub genes (*RPS27A, MAPK1, STAT1, TLR4, RPS6, FBL, TLR2, RPS3, RPS2,* and *GART*) showed excellent diagnostic value for peripheral blood with PAH and controlled peripheral blood cells (Figure 9A). In addition, we validated the ROC curve of the top ten hub genes in the GSE38267. Interestingly, the top ten hub genes showed a similar trend (Figure 9B) in integrated datasets.

### Expression validation of top ten hub genes in an independent cohort of pulmonary arterial hypertension

**FIGURE 10:**
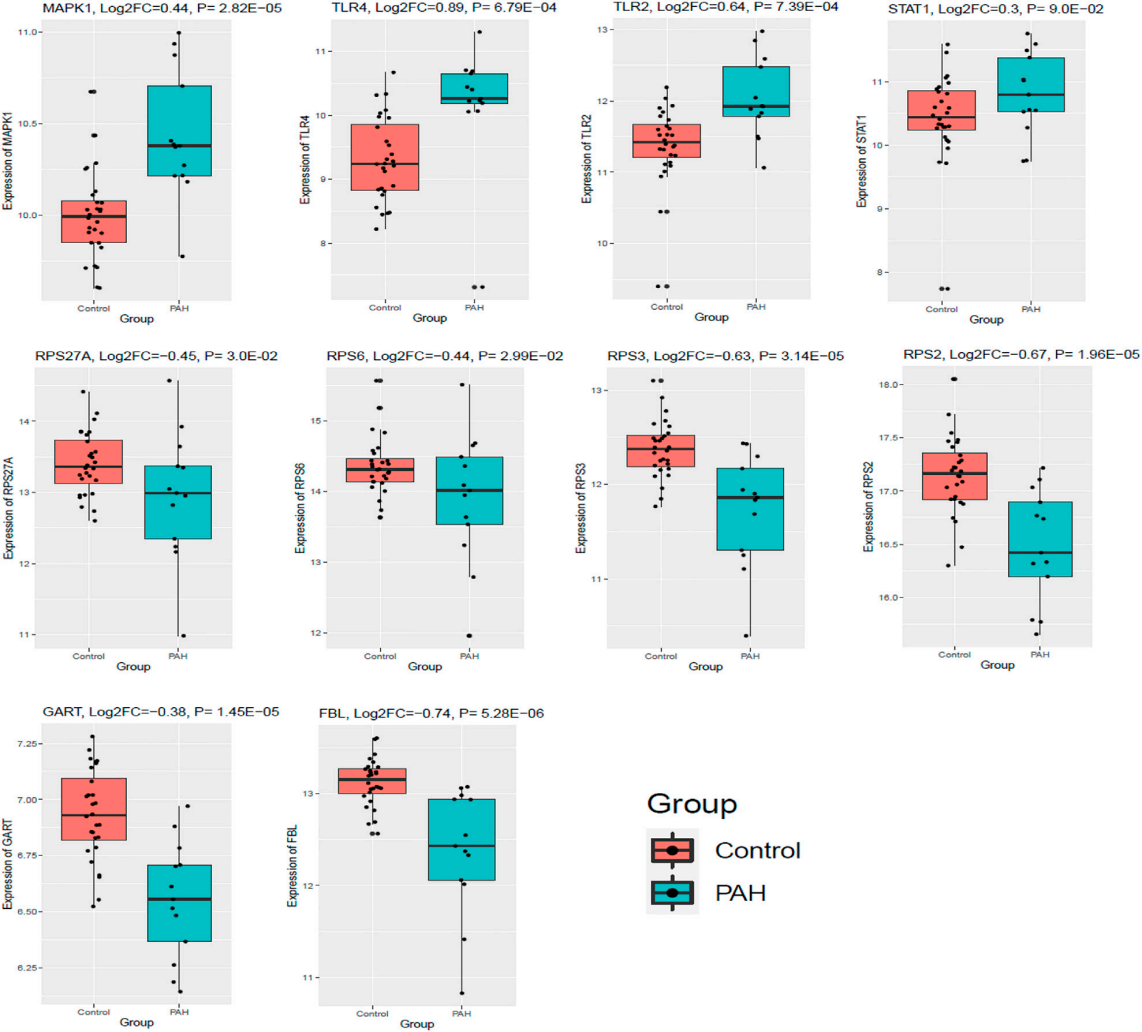
The top ten hub genes were expressed in an independent cohort of blood with PAH. GSE38267 (*n* = 41) was utilized to validate the expression of the top ten hub genes. Data were normalized into the Log2 transformation.

Finally, we validated the expression levels of the top ten hub genes in an independent cohort (GSE38267, n = 41) of the blood of PAH patients (Chesné et al., 2014). Our analysis revealed that three up-regulated top hub genes (*MAPK1, TLR4,* and *TLR2*) were also up-regulated in the blood of patients with PAH (*p* < 0.05). In addition, all six down-regulated hub genes (*RPS27A, RPS6, FBL, RPS3, RPS2,* and *GART*) are consistently down-regulated in the blood of patients with PAH (*p* < 0.05). However, the expression level of *STAT1* is not significantly (logFC = 0.37, *p* = 0.09) altered in an independent cohort of the blood of patients with PAH (Figure 10). These results demonstrated that blood-derived transcriptomes might have a significant contribution to PAH.

### Validation of hub genes expression in lung tissue of rats with pulmonary arterial hypertension

**FIGURE 11:**
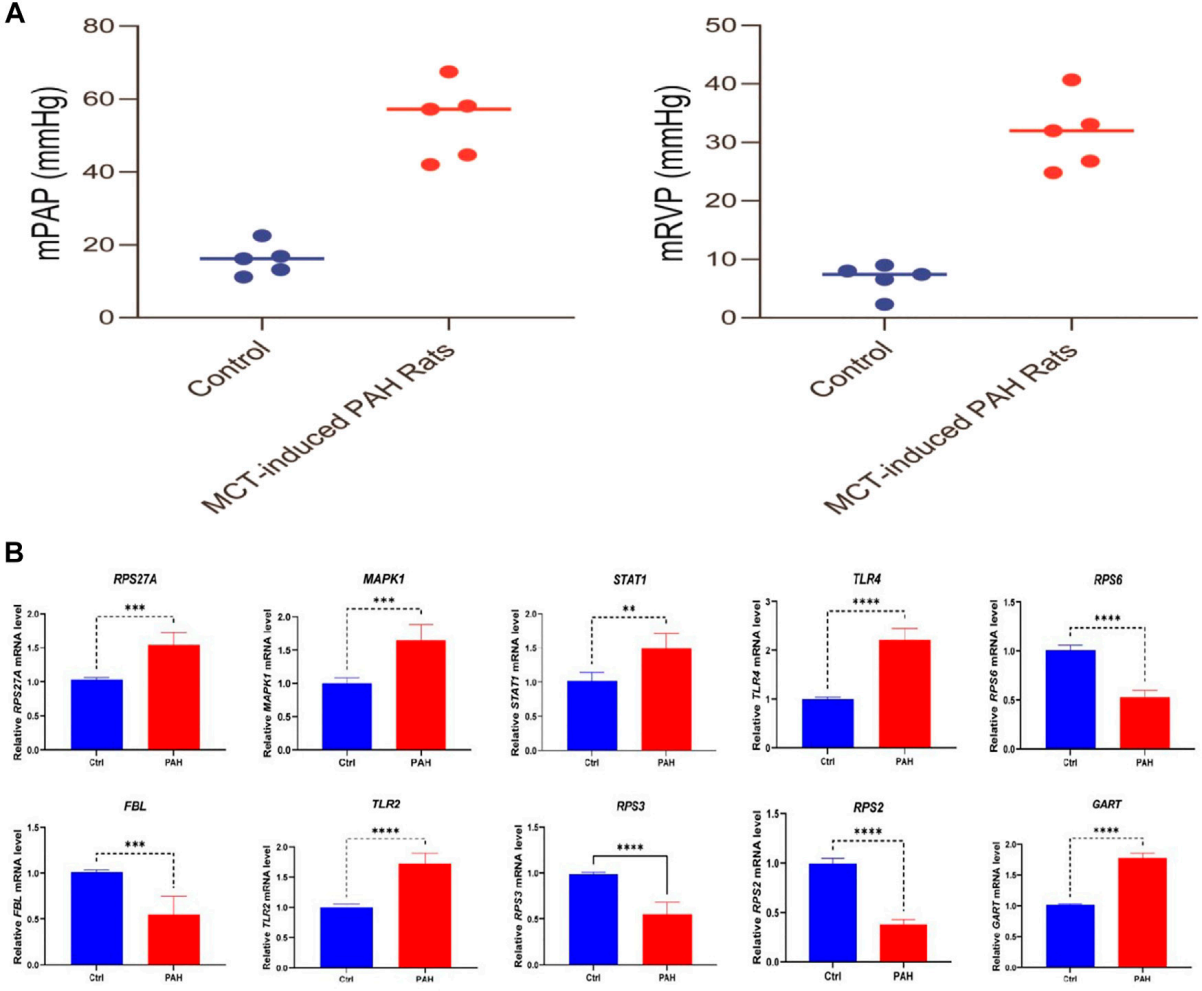
Validation of hub genes expression level in the lung tissue between rats with PAH (*n* = 5) and normal (*n* = 5). **(A)** The result of hemodynamic indicators between the control and PAH groups. **(B)** The results show that 5 screened hub genes (*MAPK1, STAT1, TLR4, TLR2, GART*) are significantly highly expressed in PAH rats, and 4 screened hub genes (*RPS6, FBL, RPS3,* and *RPS2*) are substantially lower expressed in rats with PAH. ^∗∗^ *p* < 0.01; ***p < 0.001; ^∗∗∗∗^ *p* < 0.0001.

To verify the characteristic expression of critical genes in PAH rat lung tissues, we have established PAH rats’ model and confirmed with the hemodynamic indexes (Figure 11A). Then we used qPCR to determine 10 identified essential genes (5 up-regulated genes *MAPK1, STAT1, TLR4, TLR2, GART*; 4 down-regulated genes *RPS6, FBL, RPS3, RPS2*) in lung tissues of PAH rats and normal rats. The results showed that these screened essential genes were significantly aberrantly expressed in rat lung tissues and were consistent with our analysis results (Figure 11B), suggesting that these 9 essential genes may play an important biological function in the pathogenesis of PAH. However, the expression level of *RPS27A* is not consistently deregulated in the MCT-induced rat model.

### Food and drug administration-approved drug-hub gene interaction and their molecular docking

**FIGURE 12:**
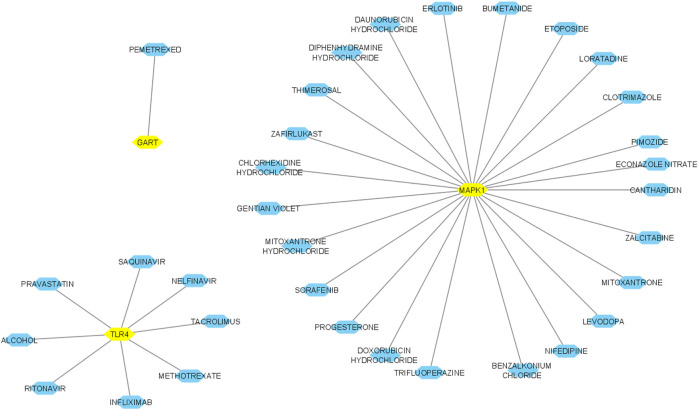
Interaction of hub genes with FDA-approved drugs. Yellow nodes indicated the hub nodes, and other nodes showed the interacting drugs. The interaction was identified and visualized using the DGIdb database and Cytoscape.

We inputted 9 validated hub genes (*MAPK1, TLR4, TLR2, RPS27A, RPS6, FBL, RPS3, RPS2,* and *GART*) into the DGIdb (Cotto et al., 2018). We found that MAPK1 interacts with identified twenty-four FDA-approved drugs (Figure 12). In addition, the eight FDA-approved drugs potentially targetting the protein product of TLR4 (Figure 12). Moreover, we revealed that the protein product of GART interacts with Pemetrexed.

**FIGURE 13:**
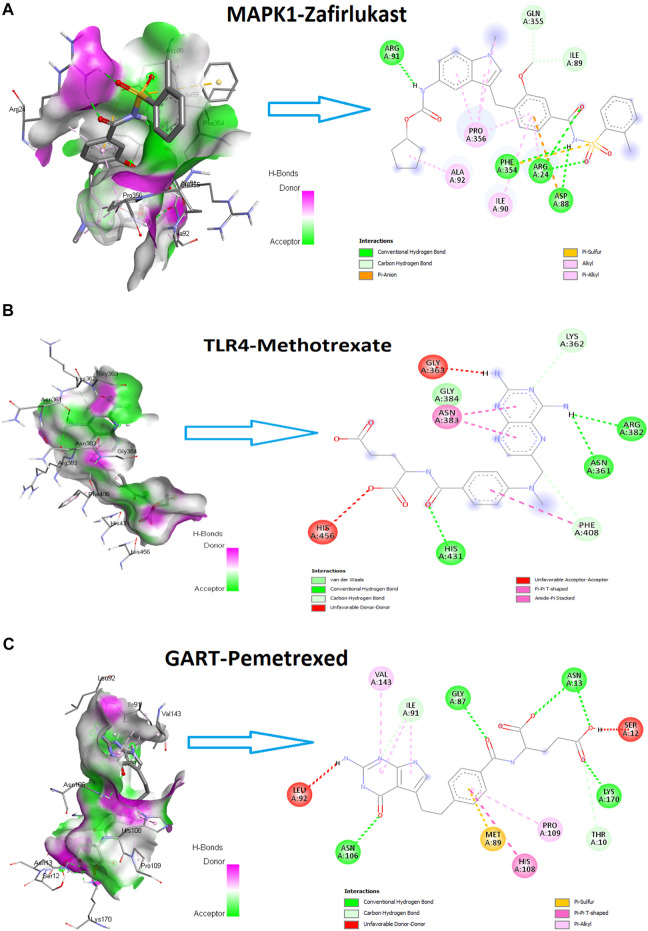
The hub proteins’ interaction (3D and 2D) with their targeting drugs. **(A).** The 3D and 2D interaction of Zafirlukast with MAPK1. The binding affinity of this interaction is −9.9. **(B).** The 3D and 2D interaction of methotrexate with TLR4. The binding affinity of this interaction is −7.7. **(C).** The 3D and 2D interaction of Pemetrexed with GART. The binding affinity of this interaction is −8.6.

After this interaction, we anticipated that these drugs could potentially interact with the amino acid residues of hub proteins with minimum binding affinity. We examined the molecular docking of protein products of these three genes (MAPK1, TLR4, and GART) to prove this hypothesis with identified drugs. Interestingly, we found that MAPK1 is potentially interacting with the 22 drugs with a minimum binding affinity (binding affinity < -5.0) (Table 7). The protein product of TLR4 is bound with methotrexate, pravastatin, and nelfinavir with a minimum binding affinity (Table 7). In addition, the GART interacts with Pemetrexed with binding affinity-8.6 (Table 7). The example interaction (3D and 2D) of hub proteins with their targeting drugs is illustrated in Figure 13 *.* The arginine, glycine, proline, isoleucine, alanine, phenylalanine, and aspartic acid residues of MAPK1 interacted with Zafirlukast (Figure 13A). Similarly, the detritus of TLR4 and GART interact with some other drugs (Figures 13B,C). Altogether, these identified drugs could be used in the clinical trial of PAH treatment.

**TABLE 7:** The binding affinity of the interacting drug with the protein product of hub genes.

Protein products	Drugs	Binding Affinity
MAPK1 (2Y9Q)	Benzalkonium	−6.4
	Bumetanide	−6.5
	Cantharidin	−6.1
	Chlorhexidine	−8.5
	Clotrimazole	−6.8
	Daunorubicin hydrochloride	−7.7
	Diphenhydramine hydrochloride	−6.8
	Doxorubicin hydrochloride	−7.3
	Econazole nitrate	−8.2
	Erlotinib	−8.6
	Etoposide	−8
	Gentian violet	−6.9
	Levodopa	−6.5
	Loratadine	−7.7
	Mitoxantrone	−8.2
	Nifedipine	−5.6
	Pimozide	−9.1
	Progesterone	−7.7
	Sorafenib	−9.3
	Trifluoperazine	−6.8
	Zafirlukast	−9.9
	Zalcitabine	−6
TLR4 (3FXI)	Methotrexate	−7.7
	Pravastatin	−5.9
	Nelfinavir	−7.3
GART (1MEN)	Pemetrexed	−8.6

## Discussion

We integrated four publicly available datasets [GSE19617 (Pendergrass et al., 2010), GSE22356 (Risbano et al., 2010), GSE33463 (Cheadle et al., 2012), and GSE131793 (Elinoff et al., 2020)] to identify the differentially expressed transcriptomic signatures in the blood of PAH patients. We identified 1,216 DEGs, including 521 up-regulated (Supplementary Table S3) and 695 down-regulated (Supplementary Table S4) in the blood of PAH patients when compared with normal samples based on combined Effect size (ES). ES is the difference between two group means divided by standard deviation, considered combinable and comparable across different studies (Xia et al., 2015a). Based on the significant transcriptomic signatures, we identified deregulated considerable gene ontology (GO) and KEGG pathways related to the pathogenesis and progression of PAH. Most of the significant-top ten biological processes included the regulation of immune systems. In addition, the up-regulated pathways were mainly involved in the regulation of the immune system, cellular development, and metabolism (Figure 2), and the down-regulated transcriptomic signatures are associated with the depressing of immune-regulation and cellular signaling pathways in PAH (Supplementary Table S8). We identified 25 MTRs that are related to the interaction of up-regulated DEGs (12 MTRs) and down-regulated DEGs (12 MTRs) in PAH (Figure 4), indicating that these transcription factors control the deregulated transcriptomic signatures in the blood of PAH patients. Soni S. Pullamsetti et al. demonstrated that the transcription factors and co-regulators are related to the expression of gene signatures, which facilitates PAH-associated vascular cell phenotypes (Pullamsetti et al., 2016). These identified TFs could be a unique target for developing novel precision medicine strategies for treating PAH.

Next, STRING and Cytoscape-based analysis identified the hub genes in the PAH. We got the 197 hub genes (the minimum degree of interaction is 25 with other DEGs). The top ten hub genes (based on the maximum degree of interaction) included *RPS27A, MAPK1, STAT1, TLR4, RPS6, FBL, TLR2, RPS3, RPS2,* and *GART* (Figure 5). We identified the 10 significant gene clusters extracted from the original PPI and investigated their association with functional enrichment. Interestingly, all of the 10 gene clusters are associated with the important (FDR<0.05) enrichment of the KEGG pathways, and the most considerable gene cluster is related to the ribosome pathway (Table 5). Also, ribosomal protein-encoding genes, such as *RPS27A, RPS6, RPS3,* and *RPS2,* are top-down-regulated hub genes in the PPI. Furthermore, the ribosome in our pathway analysis is the most down-regulated KEGG pathway (top FDR) (Supplementary Table S13). These results indicate that the ribosomal protein-encoding genes and ribosome pathway are crucial PAH regulators. Yao et al. (2018) discovered that the deregulated ribosomal proteins are restored after treating rats with PAH, indicating that ribosomes should be a key target for treating PAH.

Moreover, we extensively investigated the correlations between the top ten hub genes (*RPS27A, MAPK1, STAT1, TLR4, RPS6, FBL, TLR2, RPS3, RPS2,* and *GART*) and the ssGSEA score of various immune signatures. Interestingly, we found that numerous immune cells correlate with these hub genes’ expression. The expression levels of *RPS27A, MAPK1, STAT1, RPS6, FBL, RPS3, RPS2, and GART* hub genes are positively correlated with the infiltrations of immature dendritic cells, monocytes, and smooth muscle cells (Spearman’s correlation test, *p* < 0.05). On the other hand, the numerous immune cells, including pDC, activated dendritic cells, macrophages, neutrophils, CD4^+^ regulatory T cells, Th1 cells, eosinophil, and mast cells, are inversely correlated with the expression level of *RPS27A, MAPK1, STAT1, RPS6, FBL, RPS3, RPS2,* and *GART* hub genes (Spearman’s correlation test, *p* < 0.05) (Figure 6). In PAH, smooth muscle cells change their phenotype and express altered sensitivity to trigger the inflammation by influencing the secretion of cytokines and chemokines (Hu et al., 2020). Under inflammatory conditions, monocytes differentiate into monocyte-derived DCs, which play a substantial role in the pathobiology and development of connective tissue disease-associated idiopathic PAH (Uden et al., 2019). DCs can contribute to the pathology of PAH by activating T-cells in producing pro-inflammatory cytokines (Uden et al., 2019). The presence of perivascular macrophages is related to the pathogenic conditions of pulmonary hypertension, and interstitial macrophage-dependent inflammation is correlated with the vascular remodeling in PAH (Florentin et al., 2018). Some other immune cells, including neutrophils, mast cells, and T lymphocytes, crucially accumulate around pulmonary vessels in PAH (Hu et al., 2020). Also, the expression of these hub genes is positively correlated with hypoxia, glycolysis, fatty acid metabolism, and oxidative phosphorylation (Figures 7, 8). Furthermore, the expression levels of *RPS27A, MAPK1, STAT1, TLR4, RPS6, FBL, RPS3, RPS2,* and *GART* are positively correlated with the blood vessel remodeling genes (Table 6), indicating that these genes are critically regulating the vasculature in PAH. Inflammatory cell infiltrations are notable characteristics of aberrant vascular remodeling in PAH (Elinoff et al., 2020). Hypoxia is related to inducing pulmonary vascular remodeling and the process of lipid mediators in PAH pathophysiology (Voelkel and Tuder, 2000). Platelet glycolytic metabolism correlates with hemodynamic severity and aberrant lung vasculature in pulmonary arterial hypertension (McDowell et al., 2020). Finally, we have verified the expression level of the 10 screened hub genes in lung tissue of rats with PAH, and the validation results are highly consistent with our analysis. This result suggests that the screened key genes are significantly differentially expressed not only in peripheral blood but also in lung tissues of animal models, suggesting that the abnormal expression of these critical genes has a vital pathomechanics role in PAH. Notably, even *RPS27A* showed opposite results in our validation samples. *RPS27A* (40S ribosomal protein S27A) is a component of the 40S subunit of the ribosome and belongs to the S27AE family of ribosomal proteins. Recent research showed that *RPS27A* could interact with *RPL11*, and loss of *RPS27A* could inhibit cell viability and cell cycle, inducing apoptosis via the RPL11-MDM2-p53 pathway in lung adenocarcinoma cells (Wu et al., 2020). Besides, the significant overexpression of *RPS27A* could enhance the chemoresistance of CML cells to imatinib by trans-activating STAT3 (Wang et al., 2016). However, several studies have identified considerable upregulation of *RPS27A* expression in the left ventricular myocardium of rats suffering from end-stage PAH (MCT-induced PAH rat model), and abnormal expression of *RPS27A* has been detected in the peripheral blood of patients with connective tissue disease-associated PAH, and its key regulatory role in the pathogenesis of PAH has been identified (Hołda et al., 2020; Tu et al., 2022). Altogether, these findings confirmed that the blood-derived hub transcriptomes are associated with infiltrations of immune cells, hypoxia, and metabolism (glycolysis and fatty acid), which in turn regulates the aberrant vascular remodeling in PAH. Furthermore, our results show that the screened essential genes are significantly differentially expressed not only in peripheral blood but also dysregulated in lung tissues of animal models, suggesting that the aberrant expression of these critical genes has an important pathomechanics role in PAH.

The major drawback of this study is that the identified essential genes and regulatory networks have not been validated by experimental analysis in a laboratory or in our clinical cohort. Thus, although our findings could provide potential biomarkers for PAH diagnosis and treatment and therapeutic targets, further experimental and clinical validation is necessary to transform these significant findings into practical application in the treatment of PAH.

## Conclusion

Identifying blood-derived key transcriptional signatures is significantly associated with immune infiltrations, hypoxia, glycolysis, and blood vessel remodeling genes. These findings may provide novel insight into PAH patients’ diagnoses and therapeutic targets.

## Data Availability

The datasets presented in this study can be found in online repositories. The names of the repository/repositories and accession number(s) can be found in the article/Supplementary Material.
